# Supplementary material for: Topographic mapping of the glioblastoma proteome reveals a triple-axis model of intra-tumoral heterogeneity
Source: Nat Commun. 2022 Jan 10;13:116. doi: 10.1038/s41467-021-27667-w (PMC8748638; doi:10.1038/s41467-021-27667-w)
Supplement: Supplementary file 1 — Supplemental Information [file 41467_2021_27667_MOESM1_ESM.pdf]

Supplementary Figures and Files

Title:

Topographic mapping of the glioblastoma proteome reveals a triple axis model of intra-tumoral heterogeneity

Author list:

K.H. Brian Lam[1], Alberto J. Leon[2], Weili Hui[2], Sandy Che-Eun Lee[2,3], Ihor Batruch[4], Kevin Faust[2,5], Almos Klekner[6], Gábor Hutóczki[6], Marianne Koritzinsky[2,3,7,8], Maxime Richer[9], Ugljesa Djuric[1,2,10], Phedias Diamandis[1-3,10]*

Affiliations:

[1]Department of Laboratory Medicine and Pathobiology, University of Toronto, Toronto, Ontario, M5S 1A8, Canada,
[2]Princess Margaret Cancer Center, University Health Network, Toronto, Ontario, 610 University Avenue, M5G 2C1, Canada
[3]Institute of Medical Science, University of Toronto, Toronto, Ontario, #2374-1 King's College Circle, M5S 1A8, Canada
[4]Department of Pathology and Laboratory Medicine, Mount Sinai Hospital, Toronto, Ontario, M5G 1X5, Canada.
[5]Department of Computer Science, University of Toronto, 40 St.George Street, Toronto, Ontario M5S 2E4, Canada
[6]Department of Neurosurgery, Faculty of Medicine, University of Debrecen, 4032 Debrecen, Hungary
[7]Department of Radiation Oncology, University of Toronto, Toronto, Ontario, #504-149 College Street, M5T1P5, Canada
[8]Department of Medical Biophysics, University of Toronto, Toronto, Ontario, M5S 1A8, Canada,
[9]Department of Pathology, Centre Hospitalier Universitaire de Sherbrooke, 3001, 12e avenue Nord, Sherbrooke, QC, J1H 5N4, Canada
[10]Laboratory Medicine Program, University Health Network, 200 Elizabeth Street, Toronto, ON, Toronto, Ontario, M5G 2C4, Canada

*To whom correspondence should be addressed: p.diamandis@mail.utoronto.ca

Supplementary Fig. 1. Quality control and outlier analysis of proteomic samples.

Supplementary Fig. 2-25. Histomorphological associations between H&E images and PCA points.

Supplementary Fig. 26. Boxplot of Pearson correlation coefficients

Supplementary Fig. 27. CD276 regional enrichment

Supplementary Fig. 28. Dimensional reduction analysis (t-SNE) using ssGSEA scores from 64 gene signatures

Supplementary Fig. 29. Comparison of the hierarchical clustering analyses

Supplementary Fig. 30. GSEA of pathway components for the MSigDB signatures

Supplementary Fig. 31. Radar plots showing the functional status of histomorphometrically defined samples.

Supplementary Fig. 32. KRAS and MYC enrichment scores of cellular identities

Supplementary Fig. 33. Relative levels of gene signature enrichment across histomorphologic groups. ssGSEA scores for the 64 selected gene signatures were z-score normalized, and for each signature, statistical differences between the mean values of IT, LE, MVP and PAN were calculated with respect to CT group.

Supplementary Fig. 34. Gene signature enrichment of KRAS, MYC targets and hypoxia across GBM/s hallmark histomorphologic niches and in whole tissue samples.

Supplementary Fig. 35. Schematic representation of the machine learning architecture for niche specific inference.

Supplementary Fig. 36. Concordance between RNA-seq and proteomics enrichment scores (ssGSEA).

Supplementary Fig. 37. Pharmacological profiling highlights sensitivities of TCGA subtypes

Supplementary Fig. 38. Spheroid images upon treatment with GSK949675

Supplementary Data File 1. Patient Data.

Supplementary Data File 2. Differential expressed gene sets

Supplementary Data File 3. Proteogenomic comparisons between gene sets

Supplementary Data File 4. Selection of 64 gene signatures informative in GBM samples

Supplementary Data File 5. Pharmacotranscriptomic profiling results

Viability was calculated by taking the average alamar blue values in each condition. Differential viability was calculated by taking the alamar blue values in one condition and subtracting the other.

Supplementary Data File 6. Functional kinome profiling

Supplementary Data File 7. Antibody list

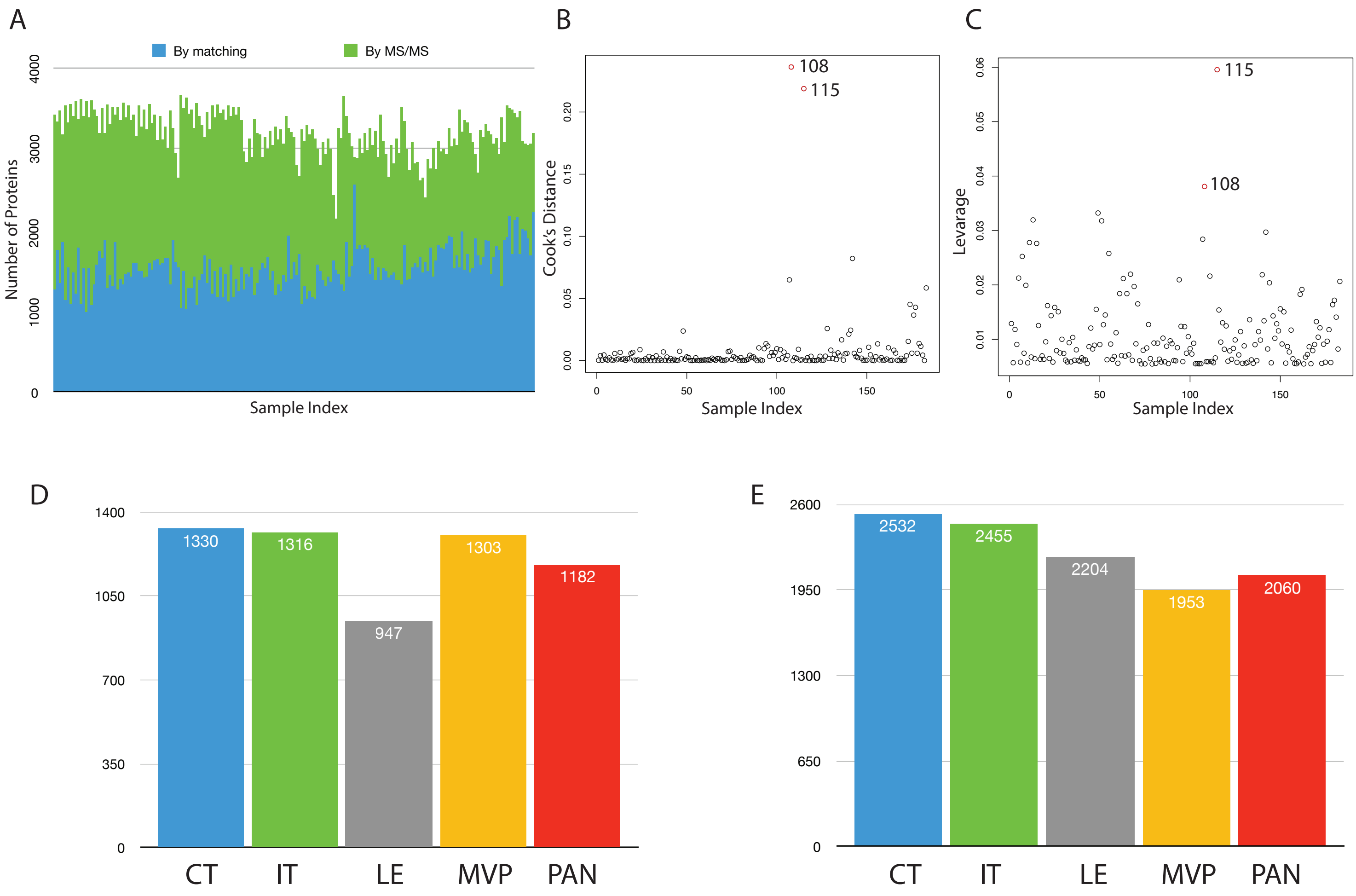

Figure S1. Quality control and outlier analysis of proteomic samples

(**A**) Stacked barchart of proteomic samples, highlighting the number of proteins identified "By matching" and "By MS/MS" from MaxQuant. Approximately 3000 proteins are identified in each sample; half the proteins are identified "by MS/MS" and "by matching". (**B**) Cook's distance analysis based on the number of proteins identified "by MS/MS" and the total number of proteins identified per sample. A cutoff at 0.1 identifies two samples as potential outliers, as highlighted in red. (**C**) A leverage analysis based on the number of proteins identified "by MS/MS" and the total number of proteins identified per sample. Similarly, this analysis also highlighted the same two samples as potential outliers. These samples were removed from further analysis. (**D**) Number of proteins identified across all samples from a specifc niche. (**E**) Number of proteins identified across atleast 60% of samples from a specific niche.

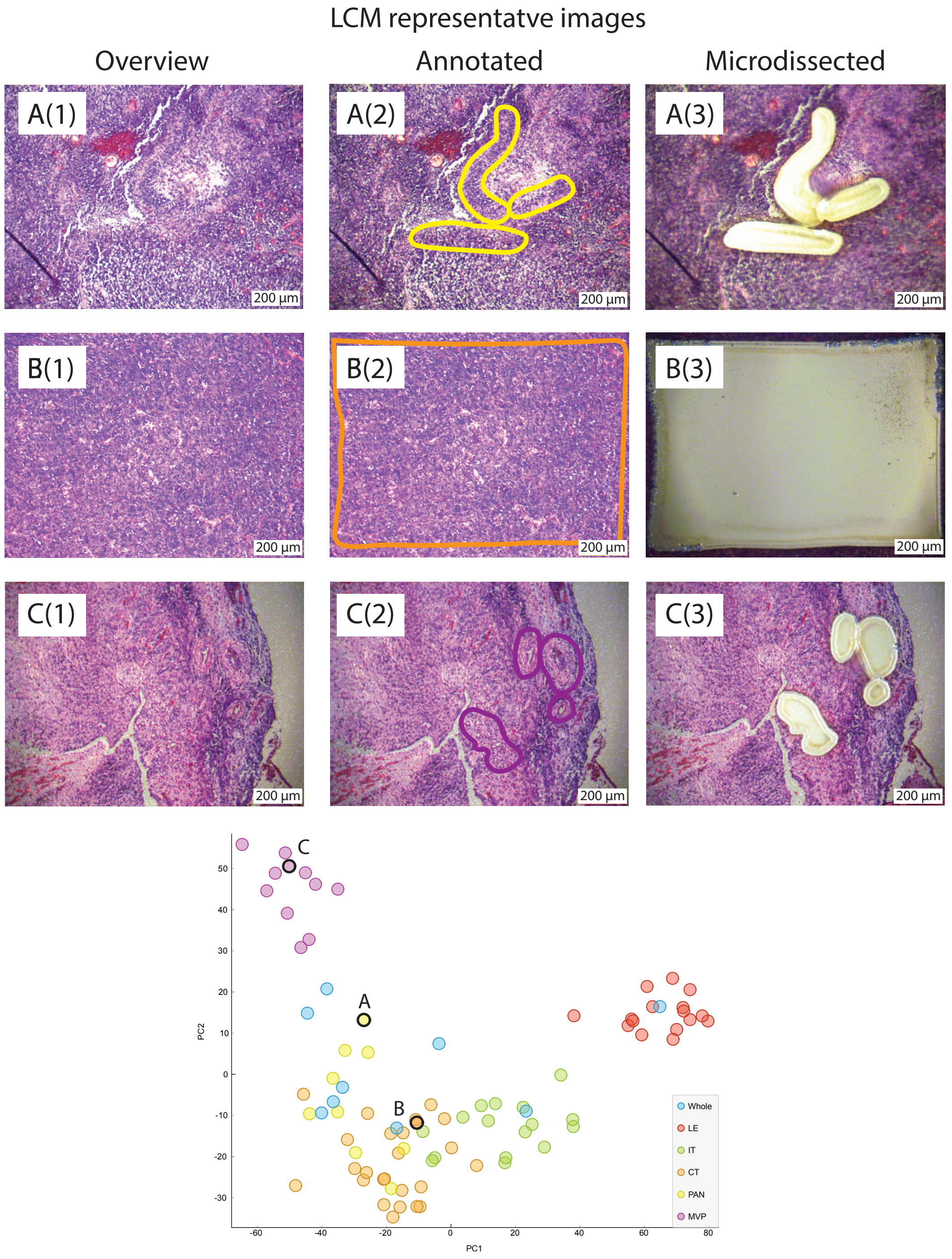

Fig S2. Patient 1 representative images before and after LCM

(**A**) PAN representative images showing the overview (1), the annotation (2) and after microdissection (3)

(**B**) CT representative images showing the overview (1), the annotation (2) and after microdissection (3)

(**C**) MVP representative images showing the overview (1), the annotation (2) and after microdissection (3)

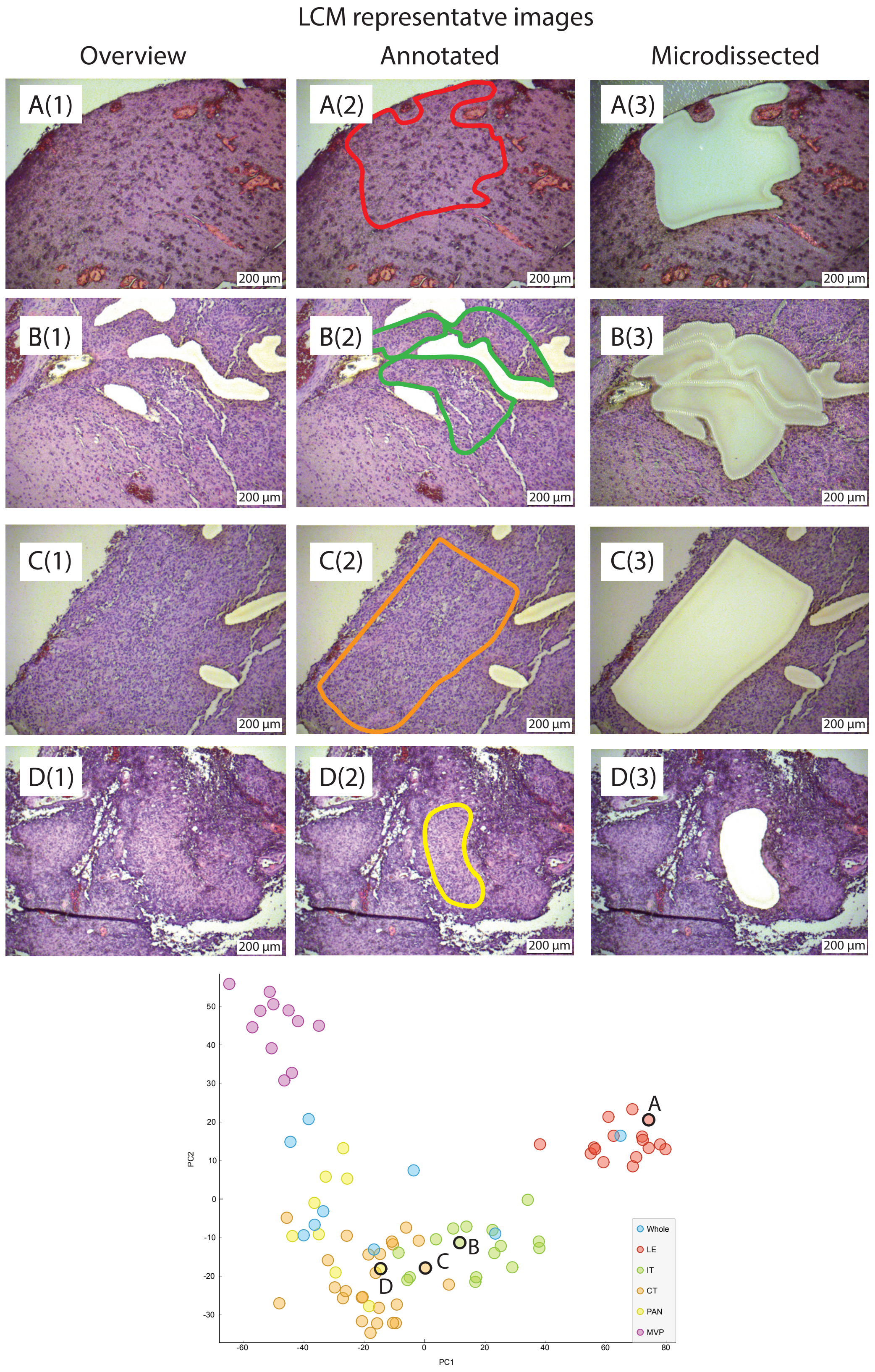

Fig S3. Patient 2 representative images before and after LMD
(**A**) LE representative images showing the overview (1), the annotation (2) and after microdissection (3)
(**B**) IT representative images showing the overview (1), the annotation (2) and after microdissection (3)
(**C**) CT representative images showing the overview (1), the annotation (2) and after microdissection (3)
(**D**) PAN representative images showing the overview (1), the annotation (2) and after microdissection (3)

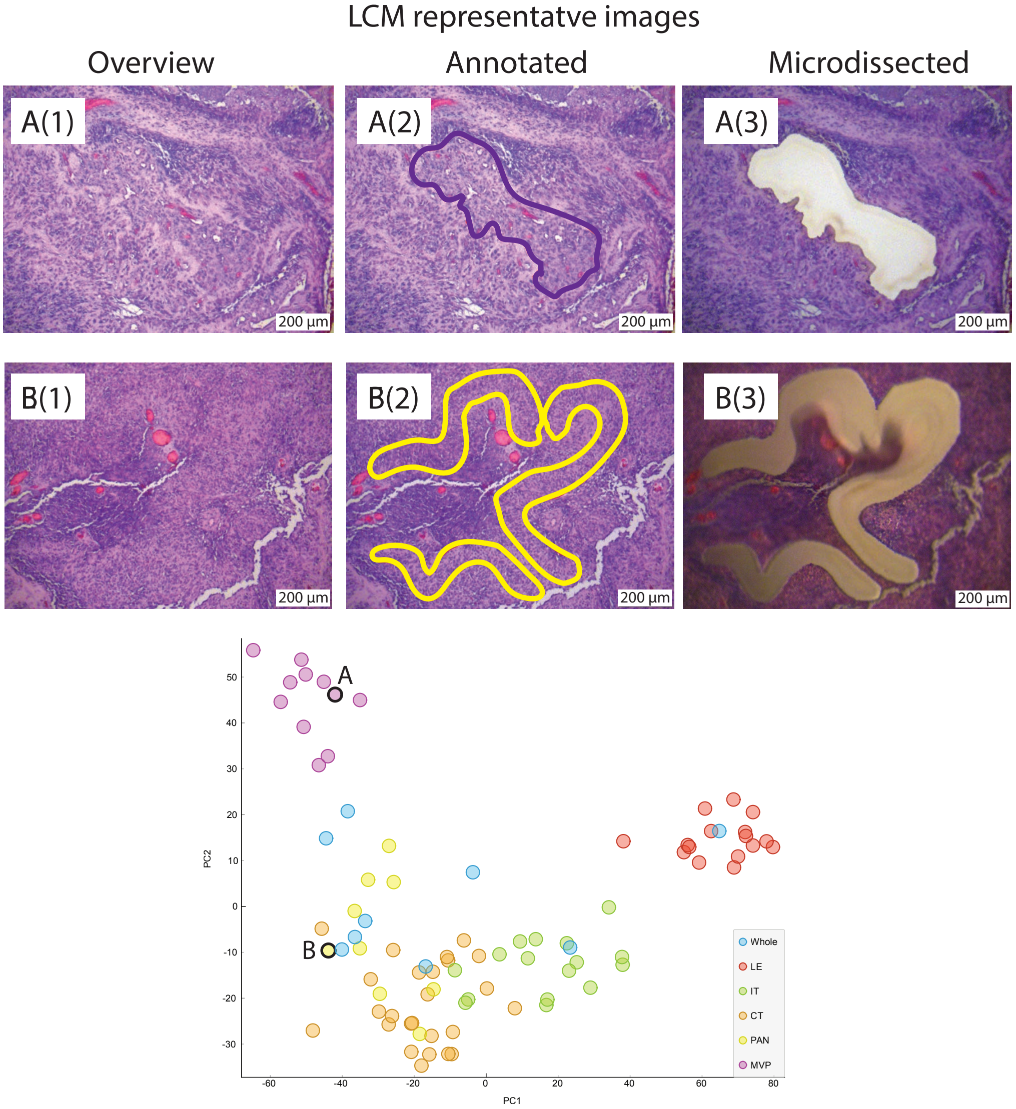

Fig S4. Patient 3 representative images before and after LMD
(**A**) MVP representative images showing the overview (1), the annotation (2) and after microdissection (3)
(**B**) PAN representative images showing the overview (1), the annotation (2) and after microdissection (3)

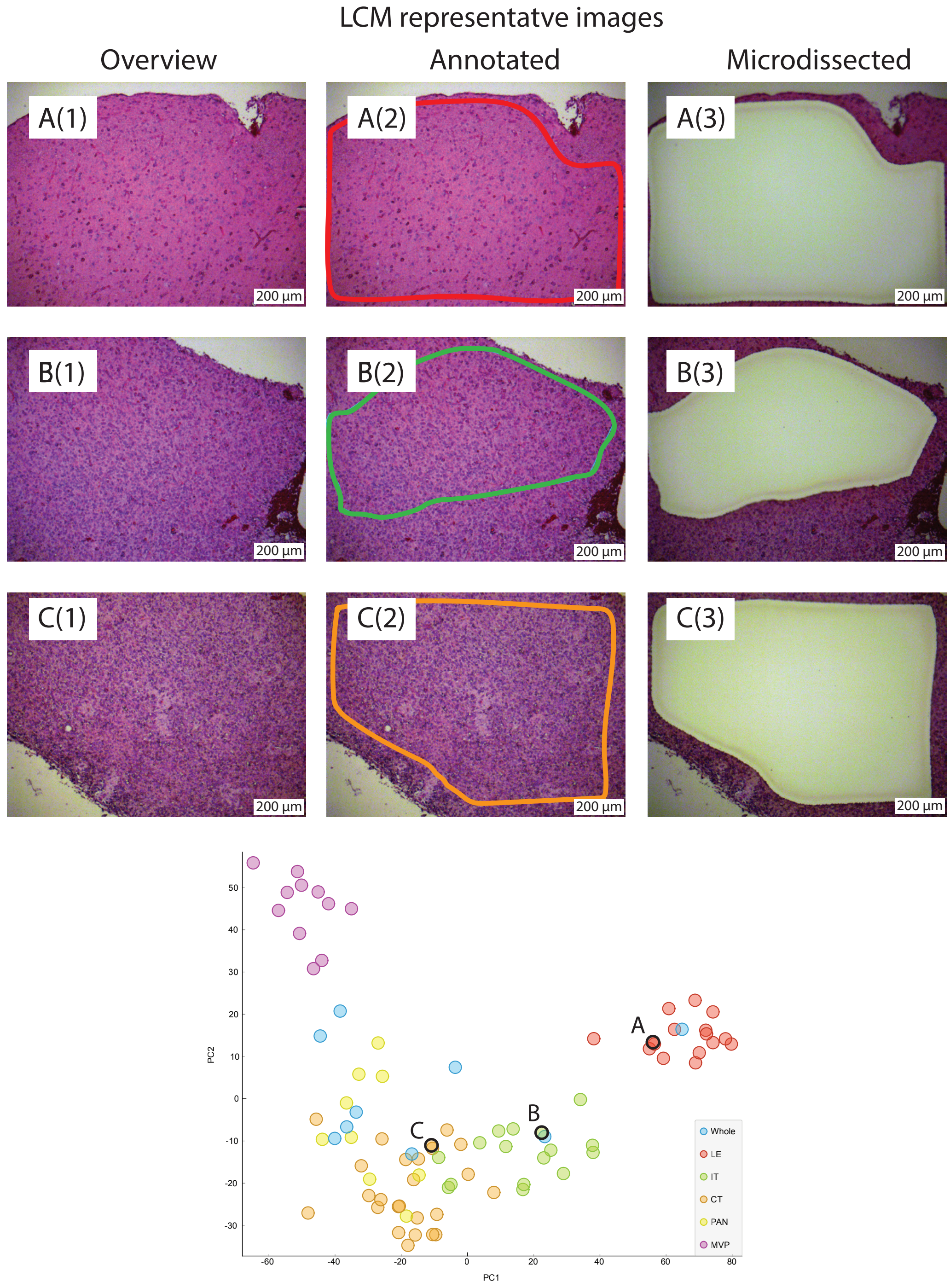

Fig S5. Patient 4 representative images before and after LMD

(**A**) LE representative images showing the overview (1), the annotation (2) and after microdissection (3)

(**B**) IT representative images showing the overview (1), the annotation (2) and after microdissection (3)

(**C**) CT representative images showing the overview (1), the annotation (2) and after microdissection (3)

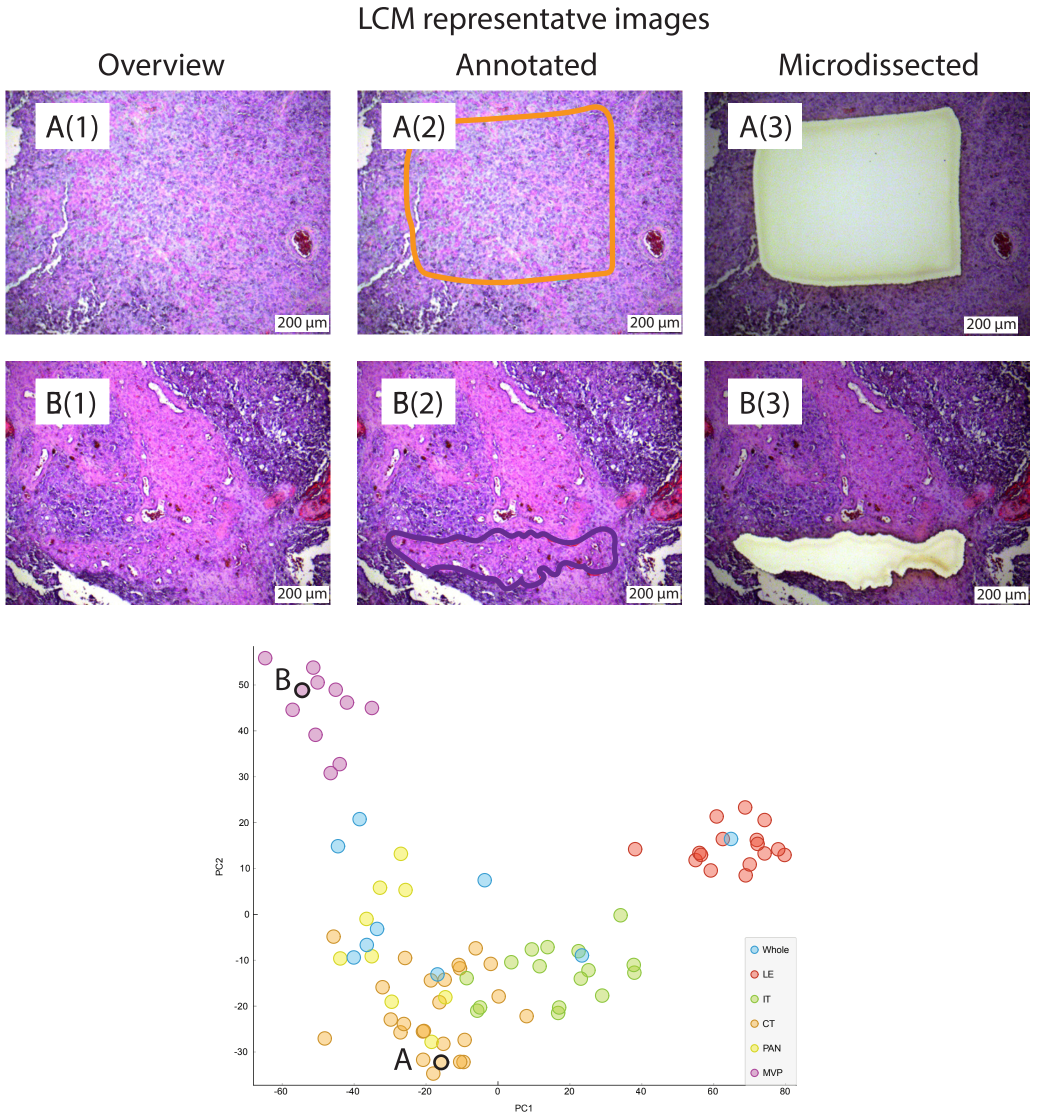

Fig S6. Patient 5 representative images before and after LMD
(**A**) CT representative images showing the overview (1), the annotation (2) and after microdissection (3)
(**B**) MVP representative images showing the overview (1), the annotation (2) and after microdissection (3)

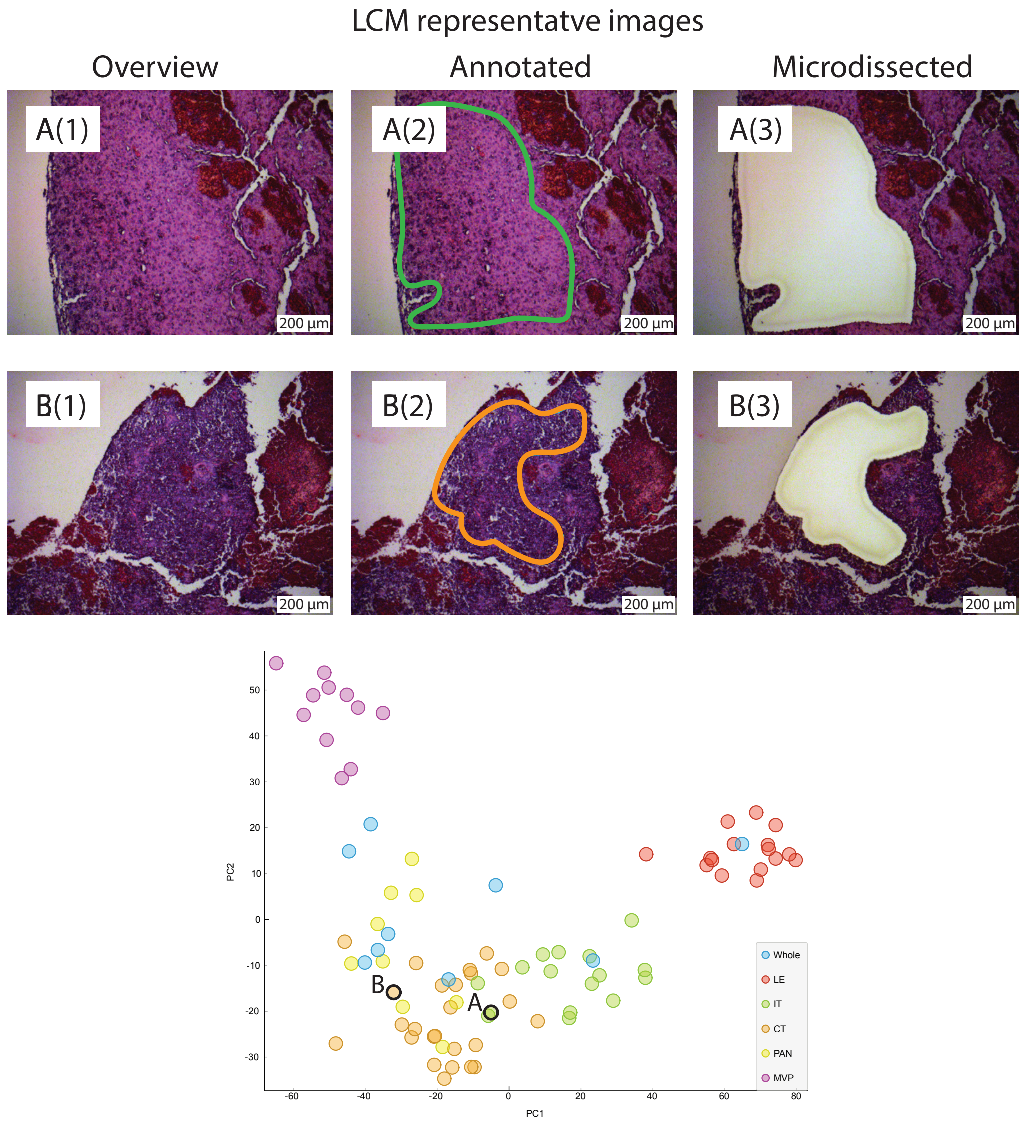

Fig S7. Patient 6 representative images before and after LMD
(**A**) IT representative images showing the overview (1), the annotation (2) and after microdissection (3)
(**B**) CT representative images showing the overview (1), the annotation (2) and after microdissection (3)

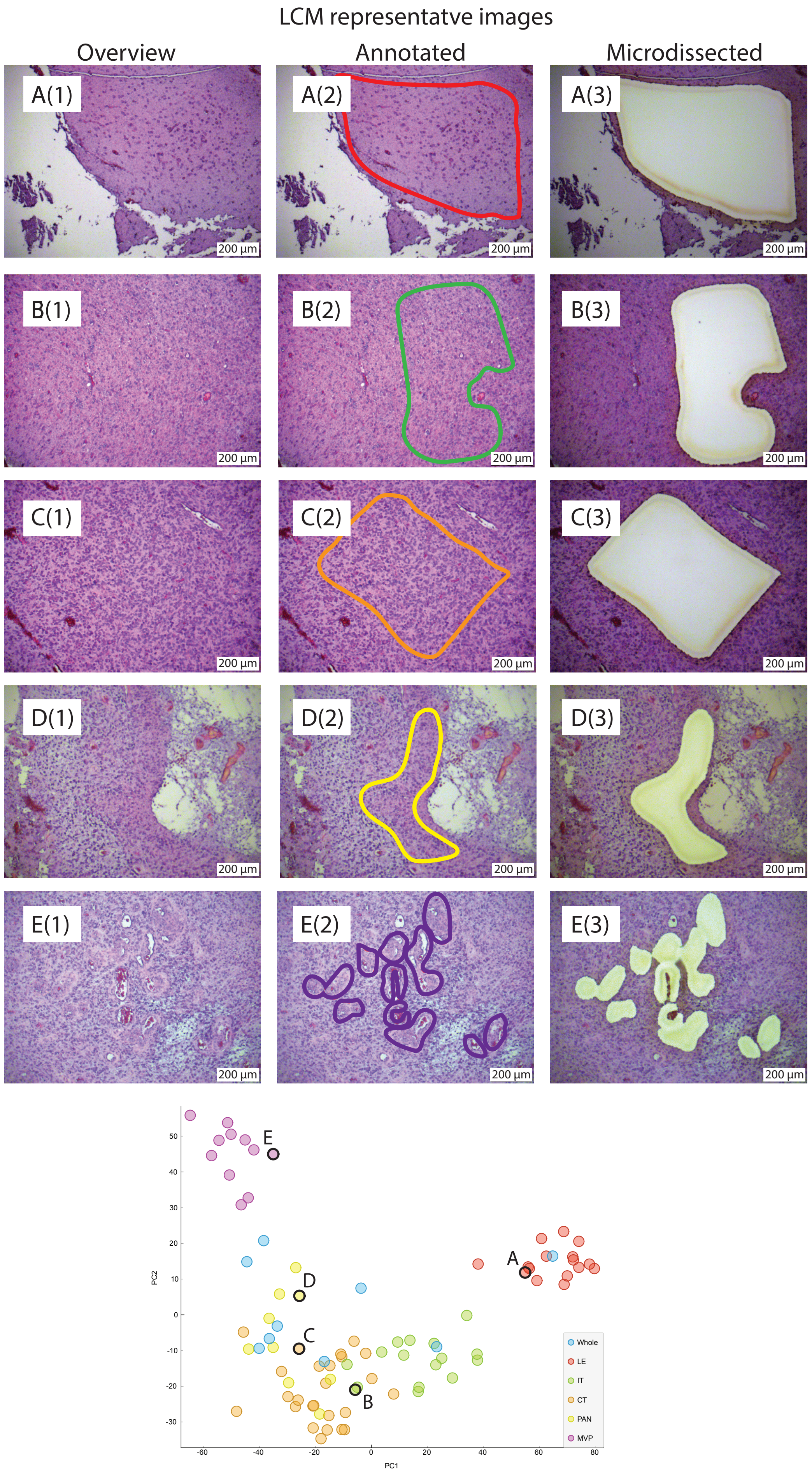

Fig S8. Patient 7 representative images before and after LMD

(**A**) LE representative images showing the overview (1), the annotation (2) and after microdissection (3)
(**B**) IT representative images showing the overview (1), the annotation (2) and after microdissection (3)
(**C**) CT representative images showing the overview (1), the annotation (2) and after microdissection (3)
(**D**) PAN representative images showing the overview (1), the annotation (2) and after microdissection (3)
(**E**) MVP representative images showing the overview (1), the annotation (2) and after microdissection (3)

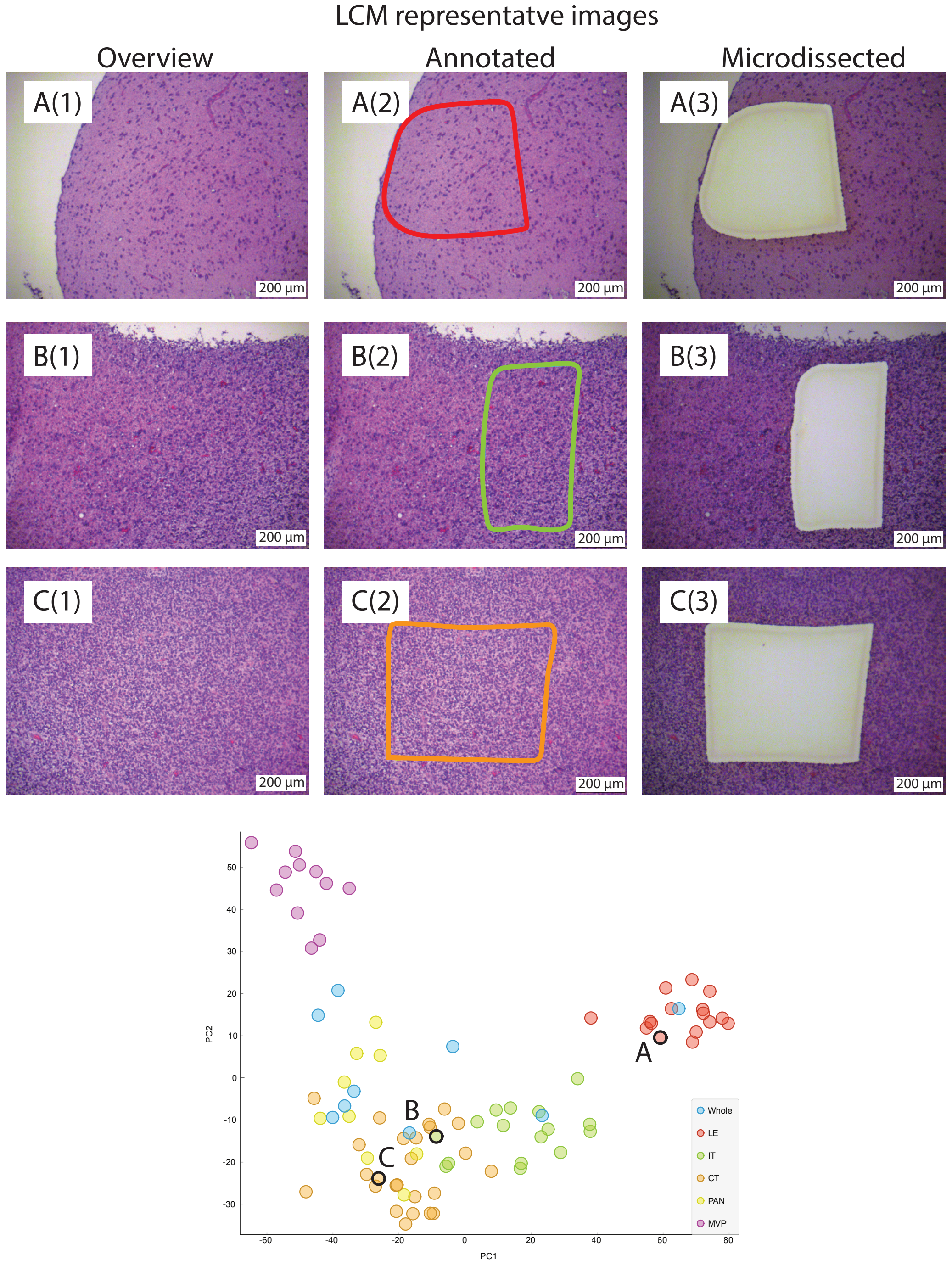

Fig S9. Patient 8 representative images before and after LMD
(**A**) LE representative images showing the overview (1), the annotation (2) and after microdissection (3)
(**B**) IT representative images showing the overview (1), the annotation (2) and after microdissection (3)
(**C**) CT representative images showing the overview (1), the annotation (2) and after microdissection (3)

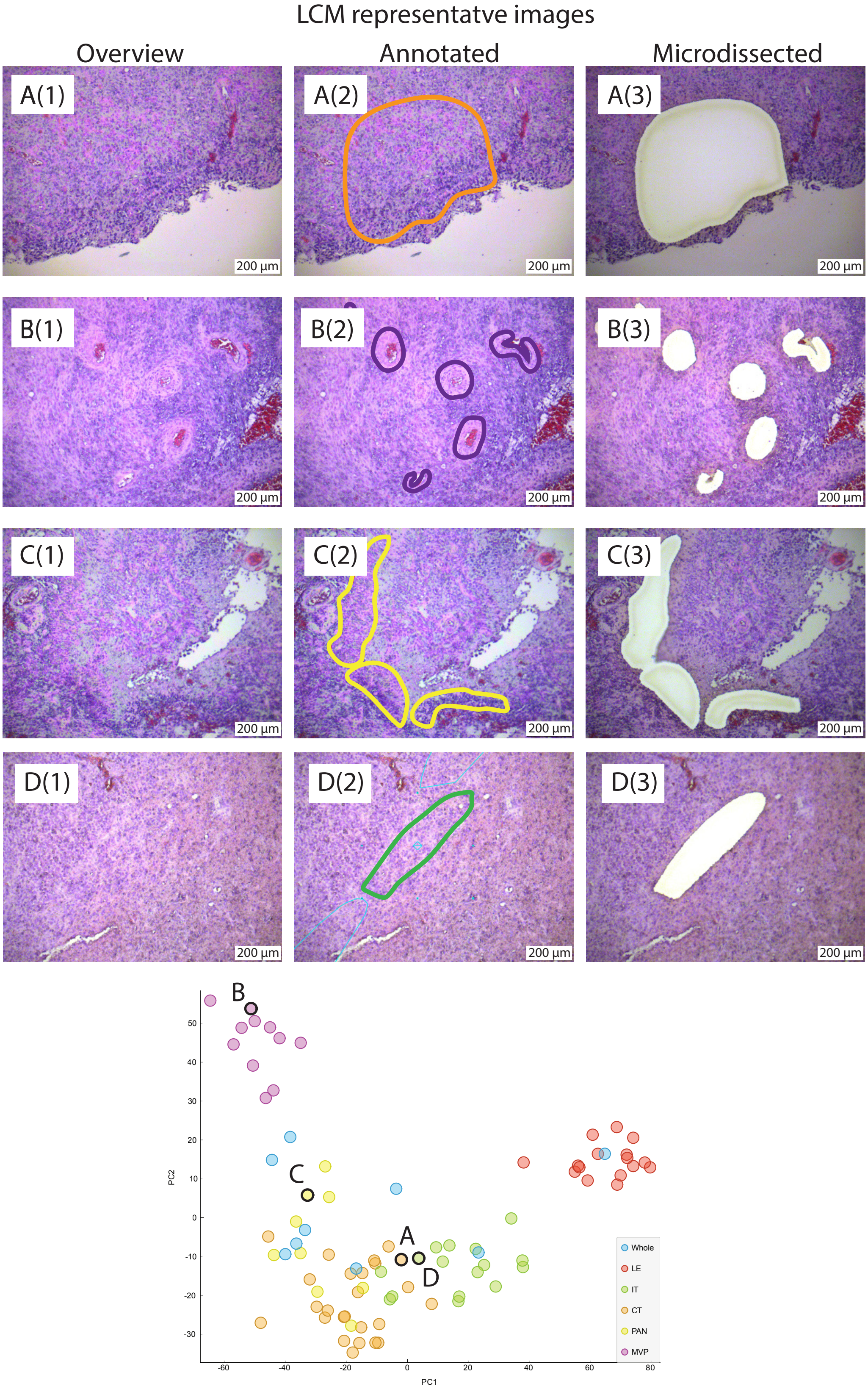

Fig S10. Patient 9 representative images before and after LMD
(**A**) CT representative images showing the overview (1), the annotation (2) and after microdissection (3)
(**B**) MVP representative images showing the overview (1), the annotation (2) and after microdissection (3)
(**C**) PAN representative images showing the overview (1), the annotation (2) and after microdissection (3)
(**D**) IT representative images showing the overview (1), the annotation (2) and after microdissection (3)

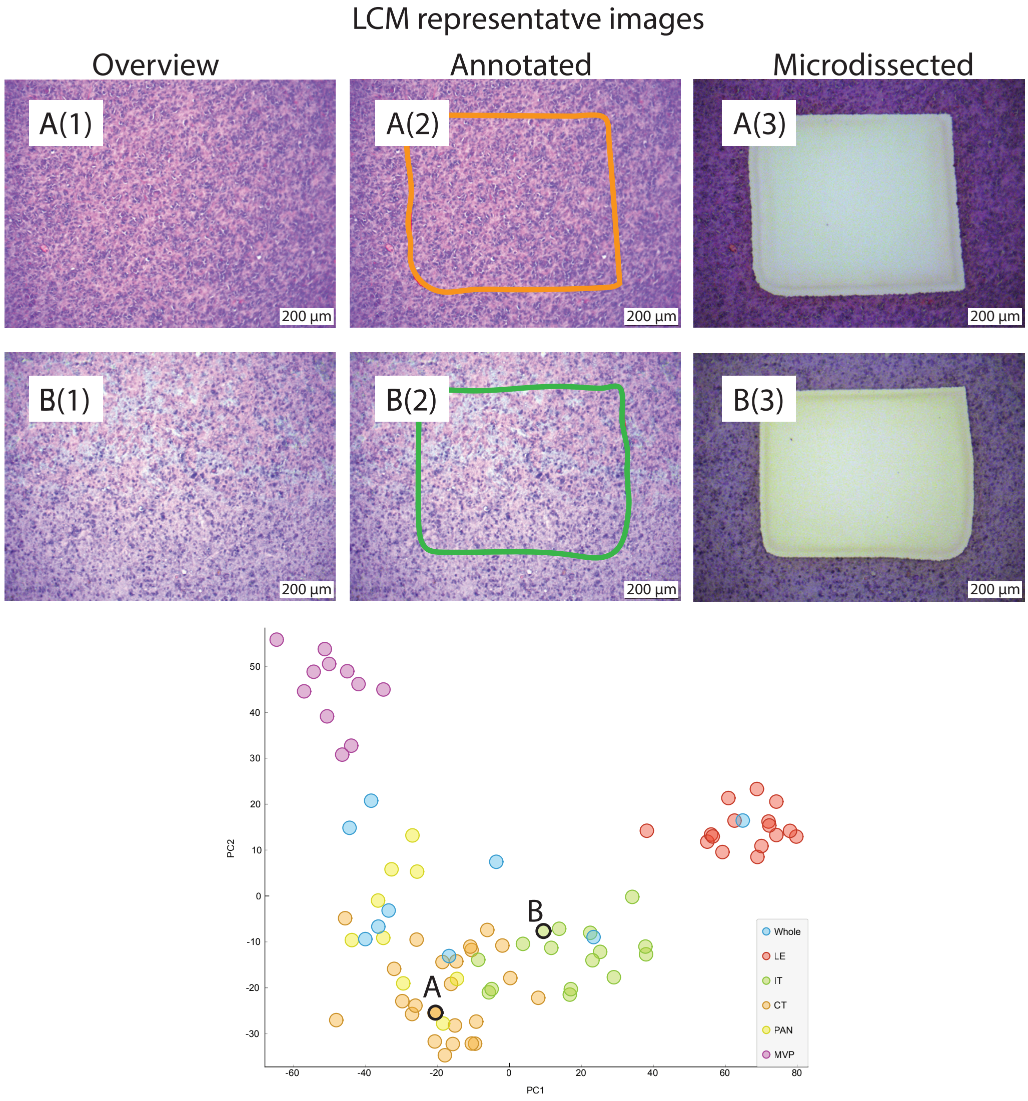

Fig S11. Patient 10 representative images before and after LMD
(**A**) CT representative images showing the overview (1), the annotation (2) and after microdissection (3)
(**B**) IT representative images showing the overview (1), the annotation (2) and after microdissection (3)

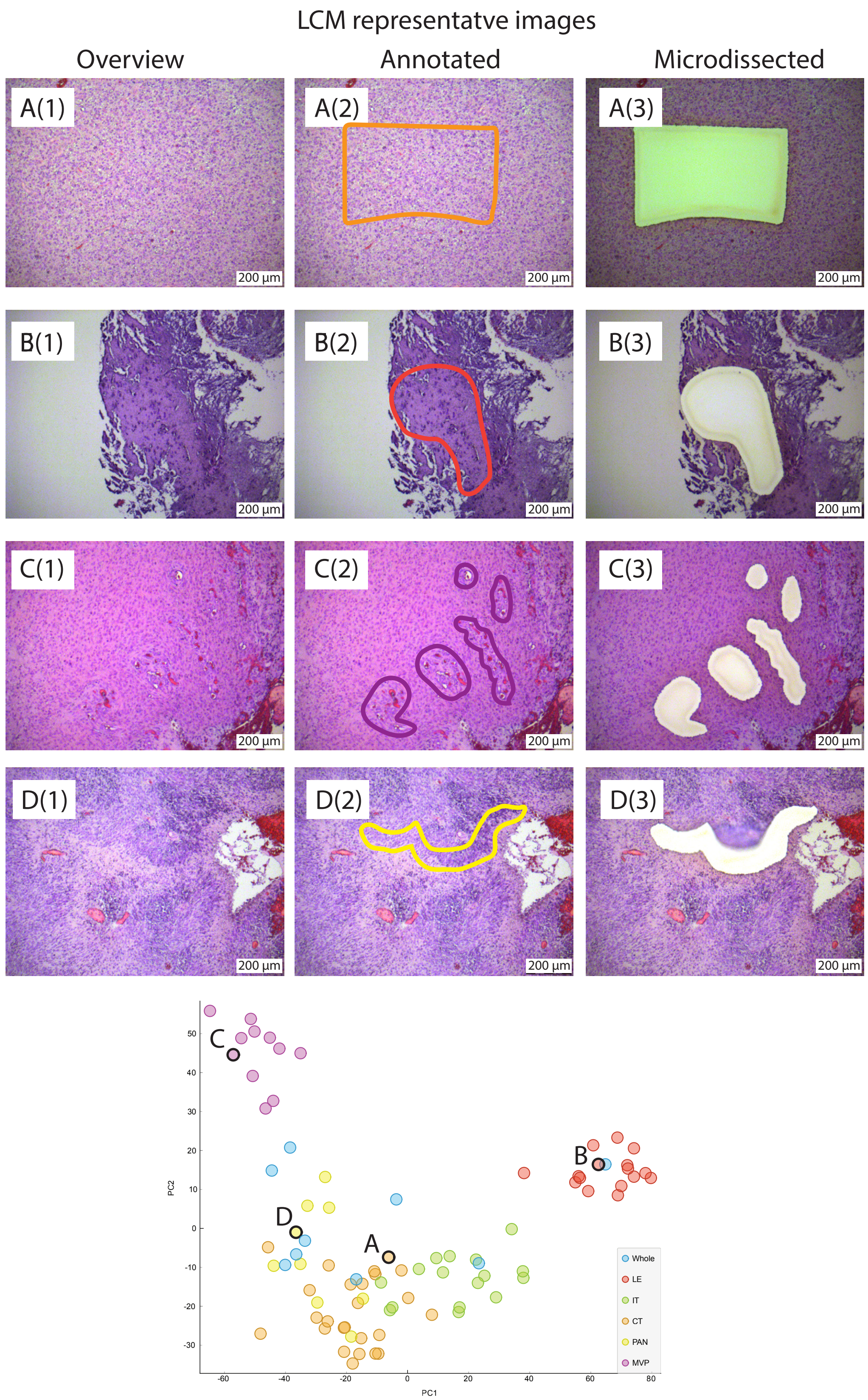

Fig S12. Patient 11 representative images before and after LMD
(**A**) CT representative images showing the overview (1), the annotation (2) and after microdissection (3)
(**B**) LE representative images showing the overview (1), the annotation (2) and after microdissection (3)
(**C**) MVP representative images showing the overview (1), the annotation (2) and after microdissection (3)
(**D**) PAN representative images showing the overview (1), the annotation (2) and after microdissection (3)

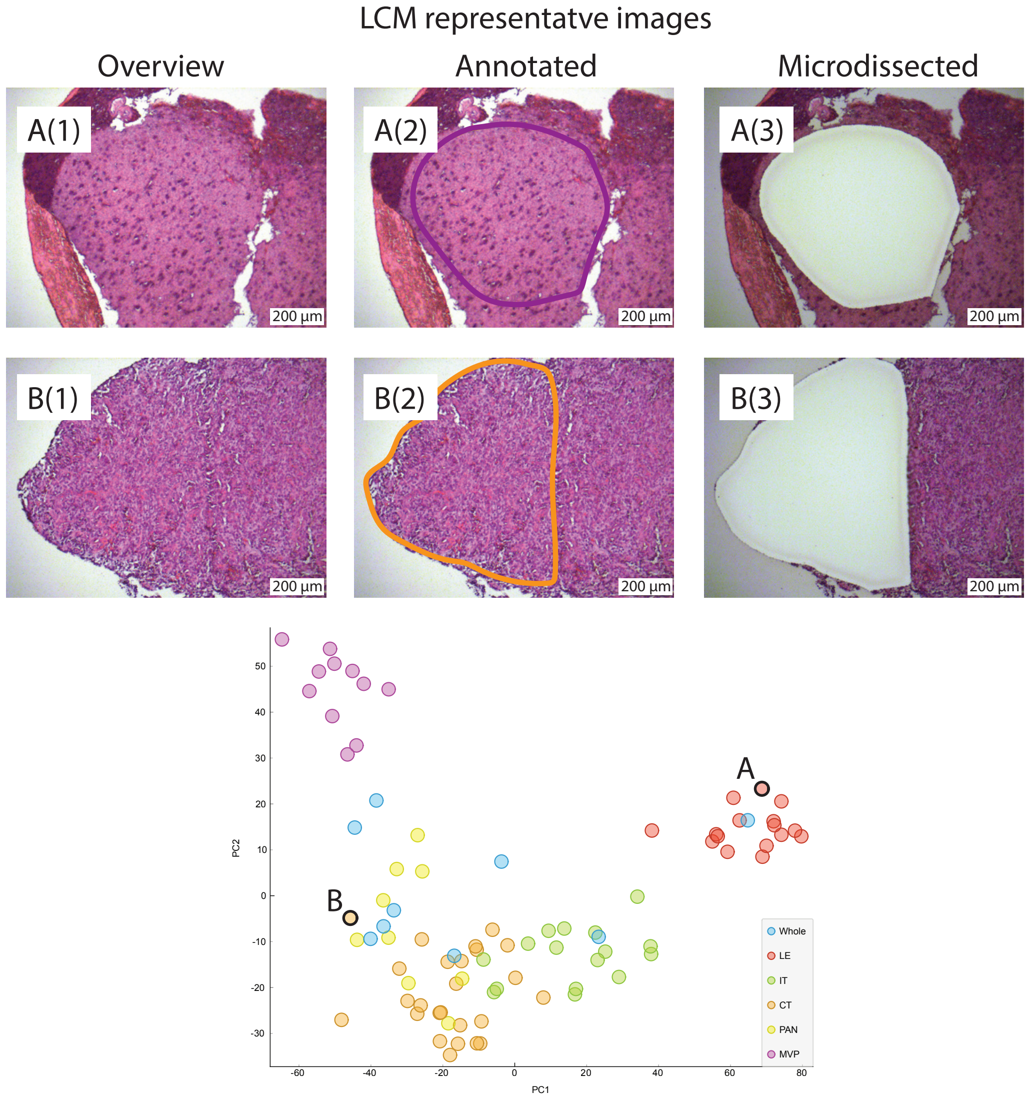

Fig S13. Patient 12 representative images before and after LMD

(**A**) LE representative images showing the overview (1), the annotation (2) and after microdissection (3)

(**B**) CT representative images showing the overview (1), the annotation (2) and after microdissection (3)

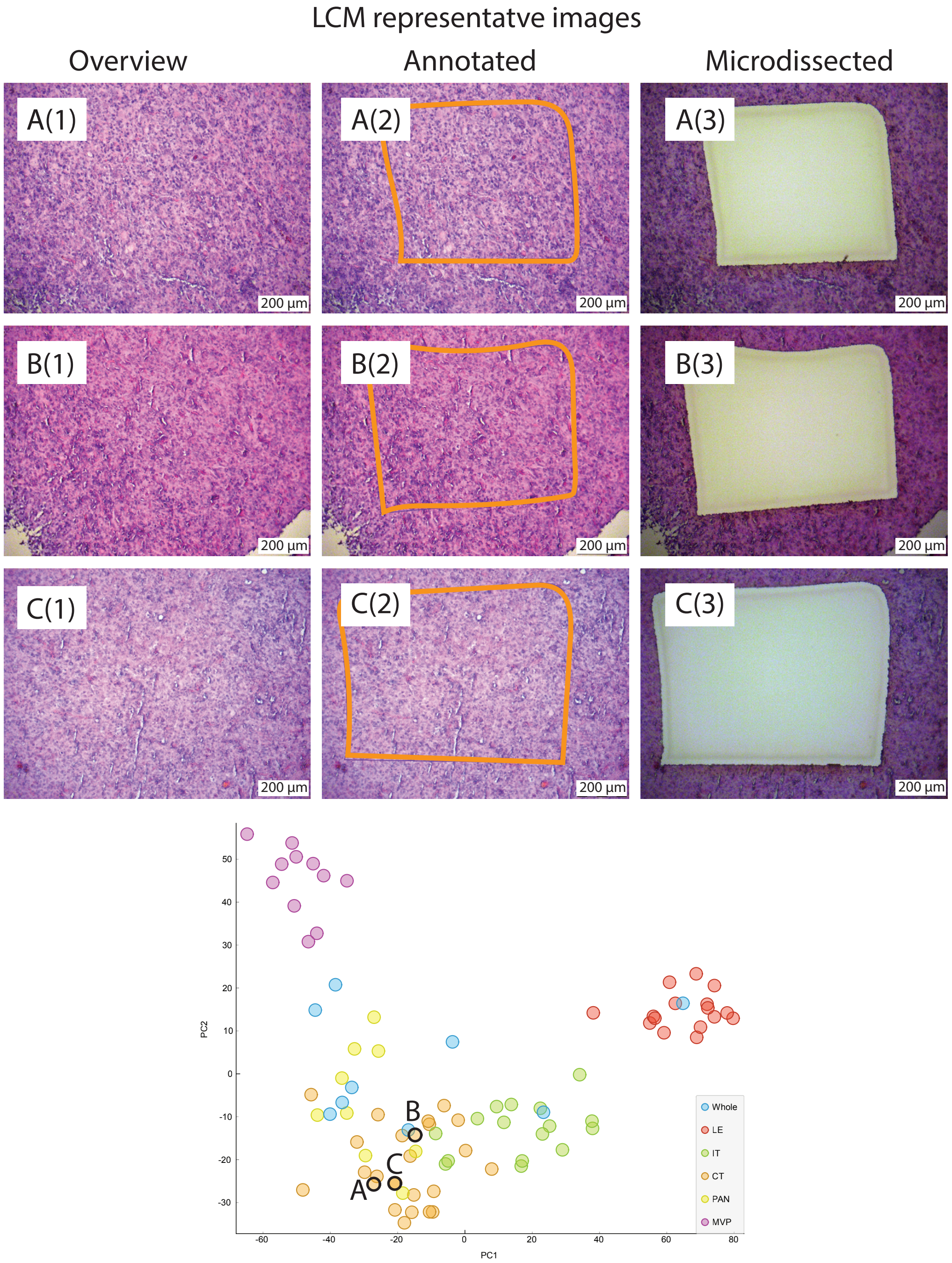

Fig S14. Patient13 representative images before and after LMD

(**A**) CT representative images showing the overview (1), the annotation (2) and after microdissection (3)

(**B**) CT representative images showing the overview (1), the annotation (2) and after microdissection (3)

(**C**) CT representative images showing the overview (1), the annotation (2) and after microdissection (3)

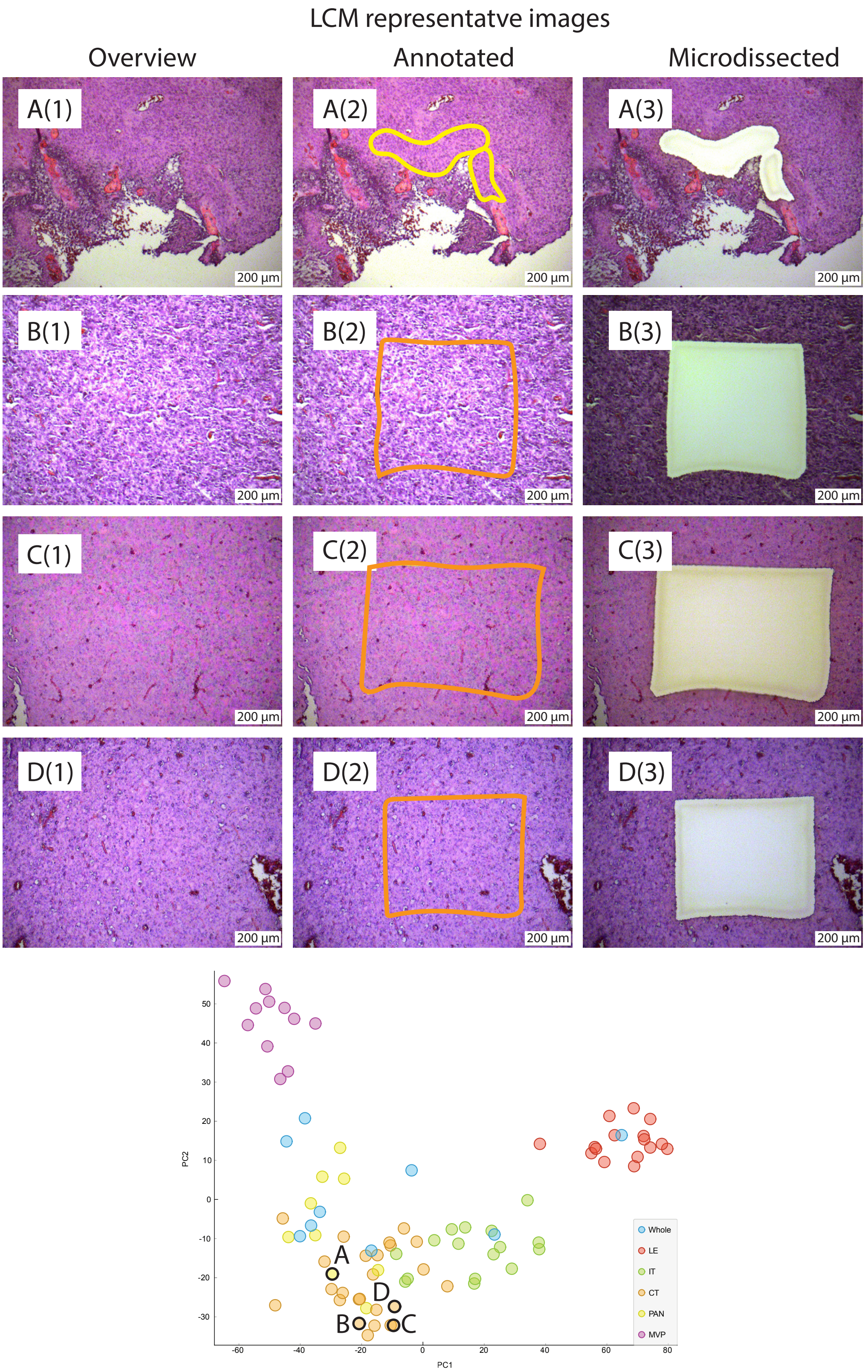

Fig S15. Patient 13 representative images before and after LMD
(**A**) PAN representative images showing the overview (1), the annotation (2) and after microdissection (3)
(**B**) CT representative images showing the overview (1), the annotation (2) and after microdissection (3)
(**C**) CT representative images showing the overview (1), the annotation (2) and after microdissection (3)
(**D**) CT representative images showing the overview (1), the annotation (2) and after microdissection (3)

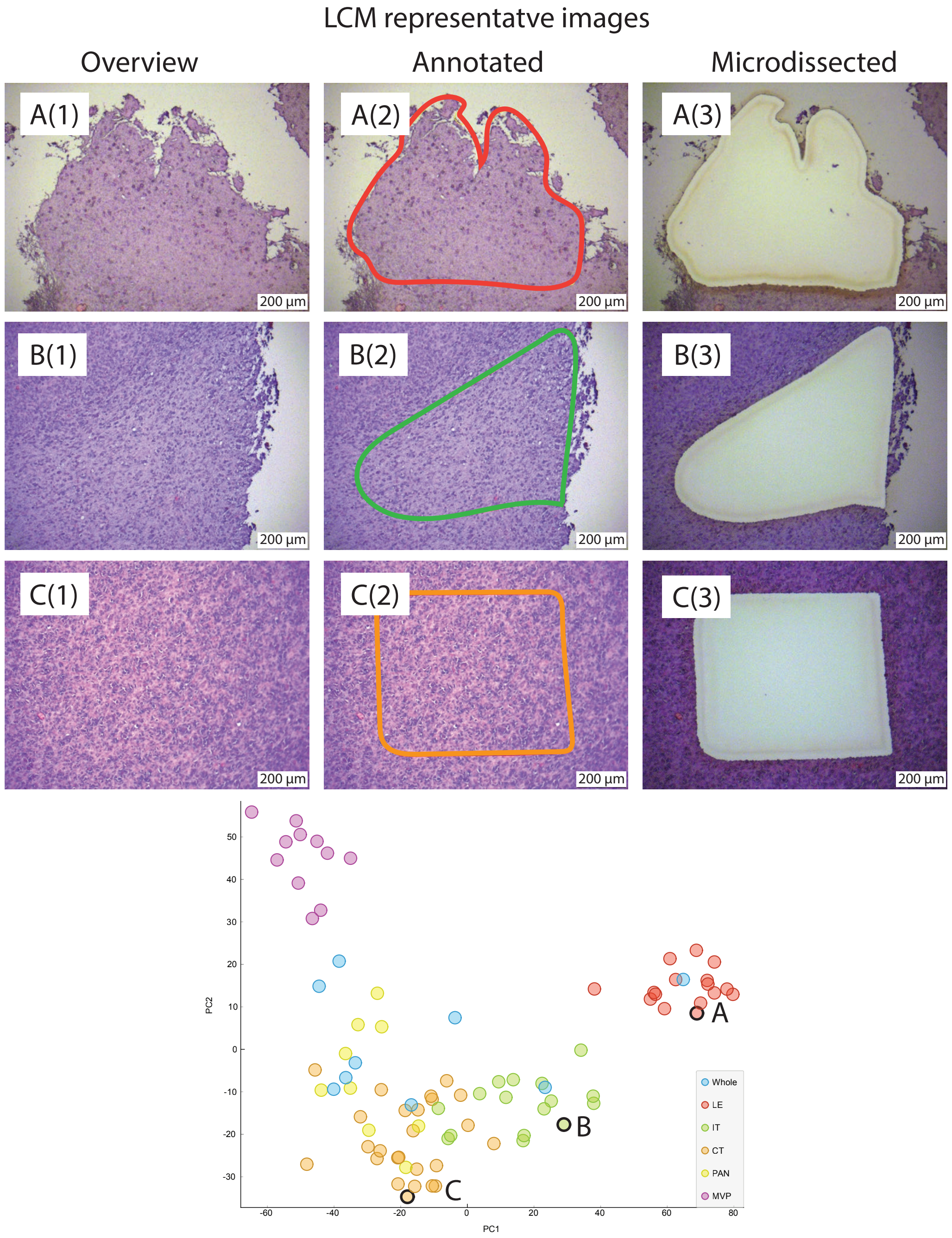

Fig S16. Patient 14 representative images before and after LMD
(**A**) LE representative images showing the overview (1), the annotation (2) and after microdissection (3)
(**B**) IT representative images showing the overview (1), the annotation (2) and after microdissection (3)
(**C**) CT representative images showing the overview (1), the annotation (2) and after microdissection (3)

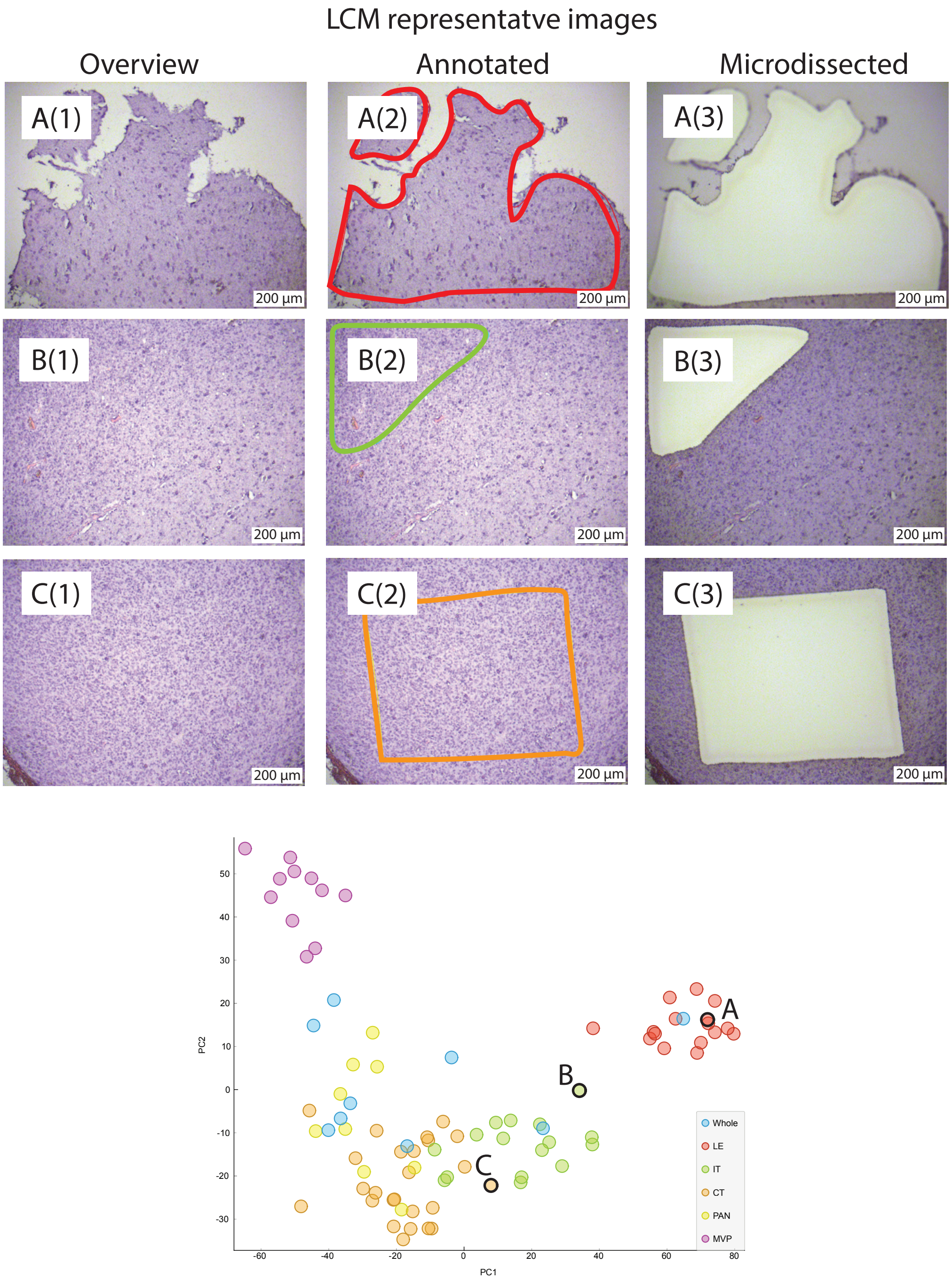

Fig S17. Patient 15 representative images before and after LMD
(**A**) LE representative images showing the overview (1), the annotation (2) and after microdissection (3)
(**B**) IT representative images showing the overview (1), the annotation (2) and after microdissection (3)
(**C**) CT representative images showing the overview (1), the annotation (2) and after microdissection (3)

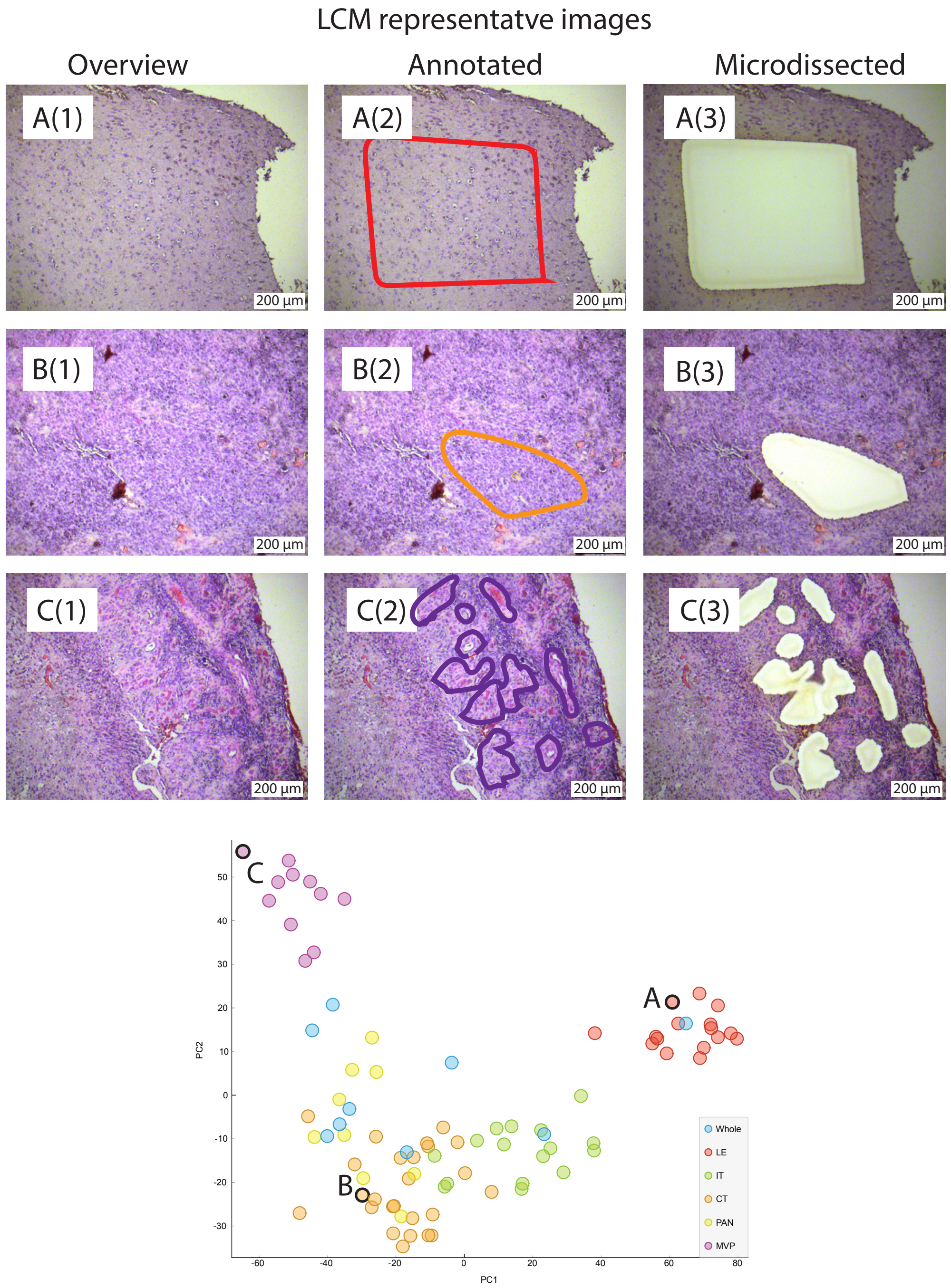

Fig S18. Patient 16 representative images before and after LMD
(**A**) LE representative images showing the overview (1), the annotation (2) and after microdissection (3)
(**B**) CT representative images showing the overview (1), the annotation (2) and after microdissection (3)
(**C**) MVP representative images showing the overview (1), the annotation (2) and after microdissection (3)

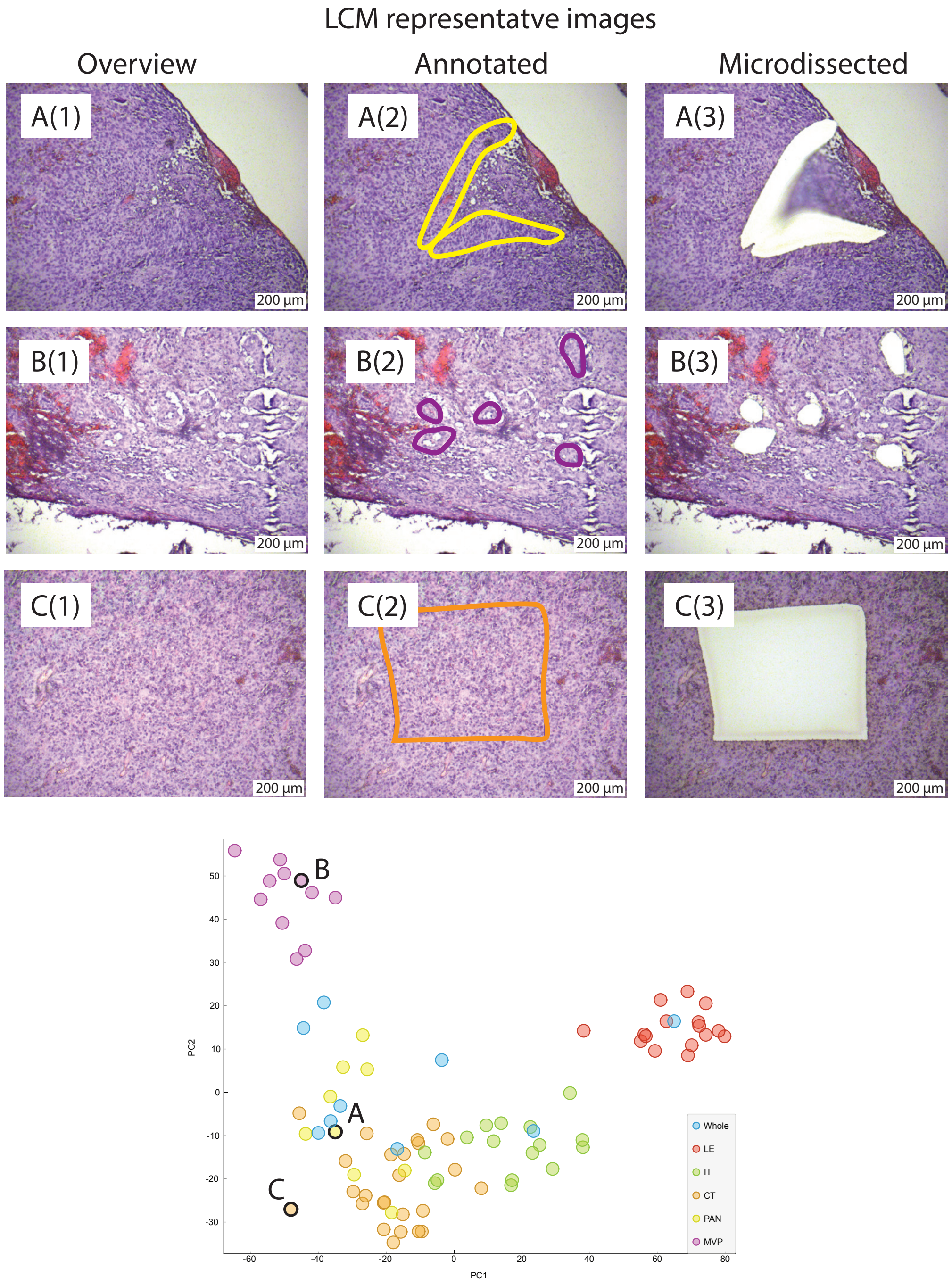

Fig S19. Patient 17 representative images before and after LMD
(**A**) PAN representative images showing the overview (1), the annotation (2) and after microdissection (3)
(**B**) MVP representative images showing the overview (1), the annotation (2) and after microdissection (3)
(**C**) CT representative images showing the overview (1), the annotation (2) and after microdissection (3)

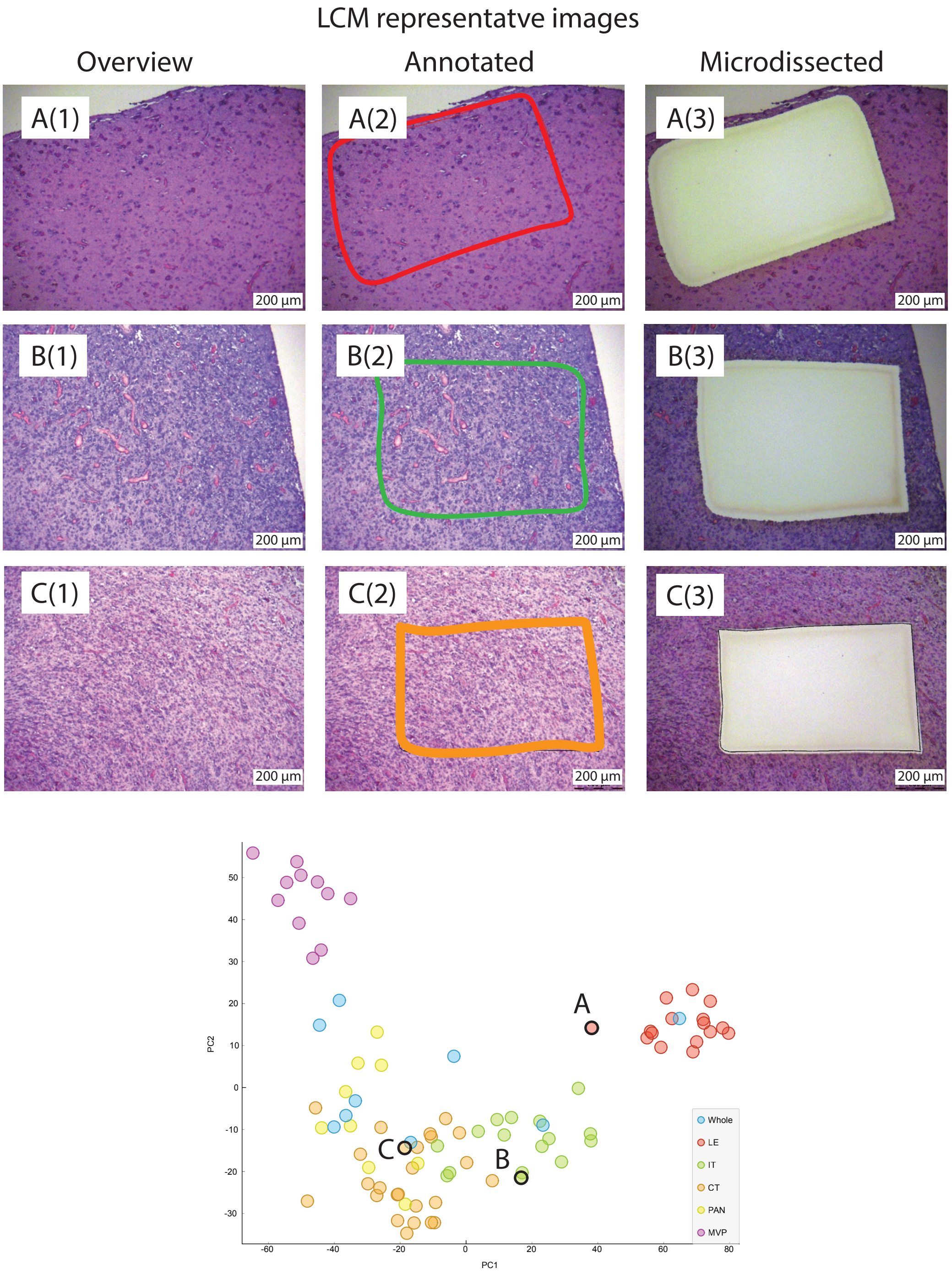

Fig S20. Patient 18 representative images before and after LMD

(**A**) LE representative images showing the overview (1), the annotation (2) and after microdissection (3)

(**B**) IT representative images showing the overview (1), the annotation (2) and after microdissection (3)

(**C**) CT representative images showing the overview (1), the annotation (2) and after microdissection (3)

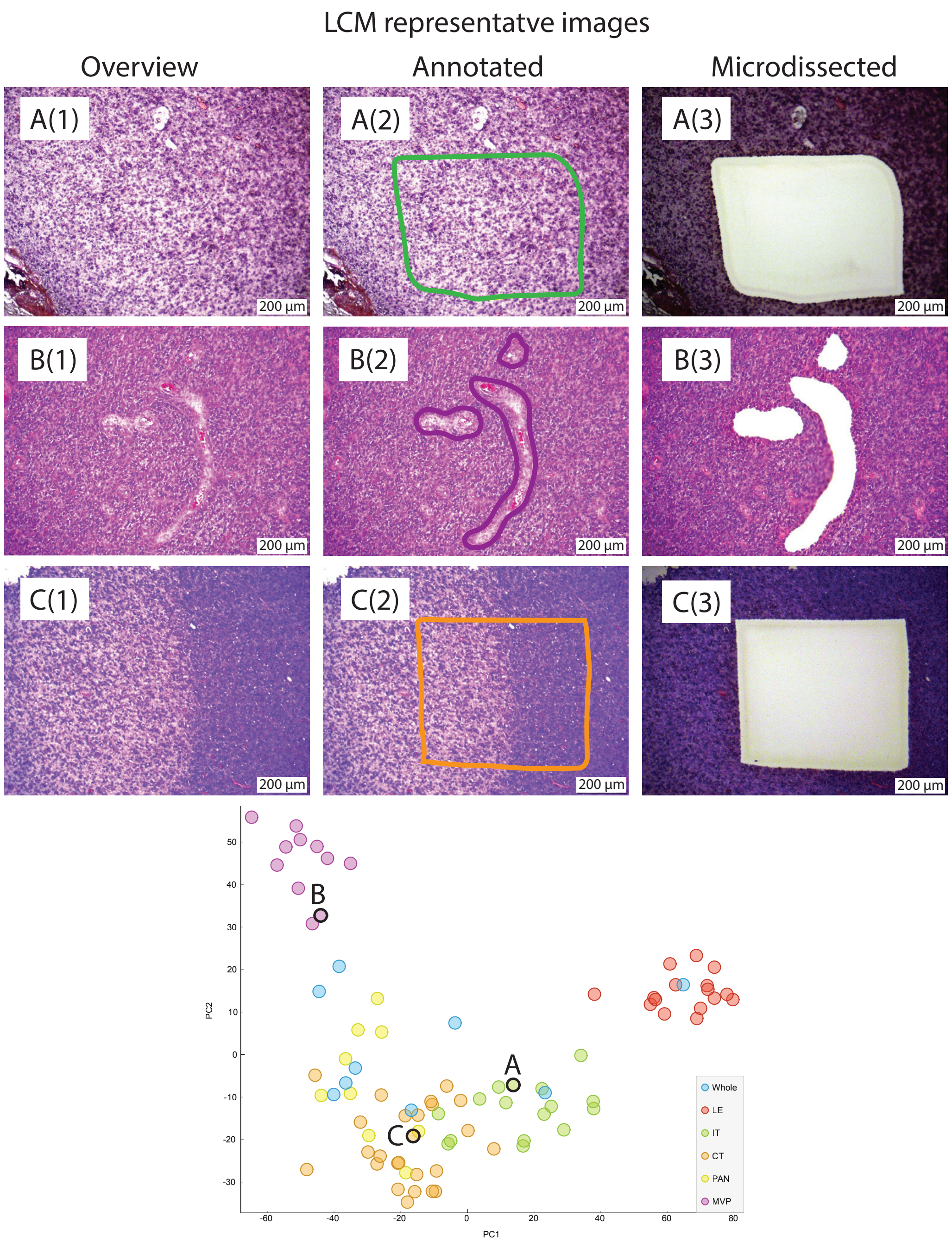

Fig S21. Patient 19 representative images before and after LMD
(**A**) IT representative images showing the overview (1), the annotation (2) and after microdissection (3)
(**B**) MVP representative images showing the overview (1), the annotation (2) and after microdissection (3)
(**C**) CT representative images showing the overview (1), the annotation (2) and after microdissection (3)

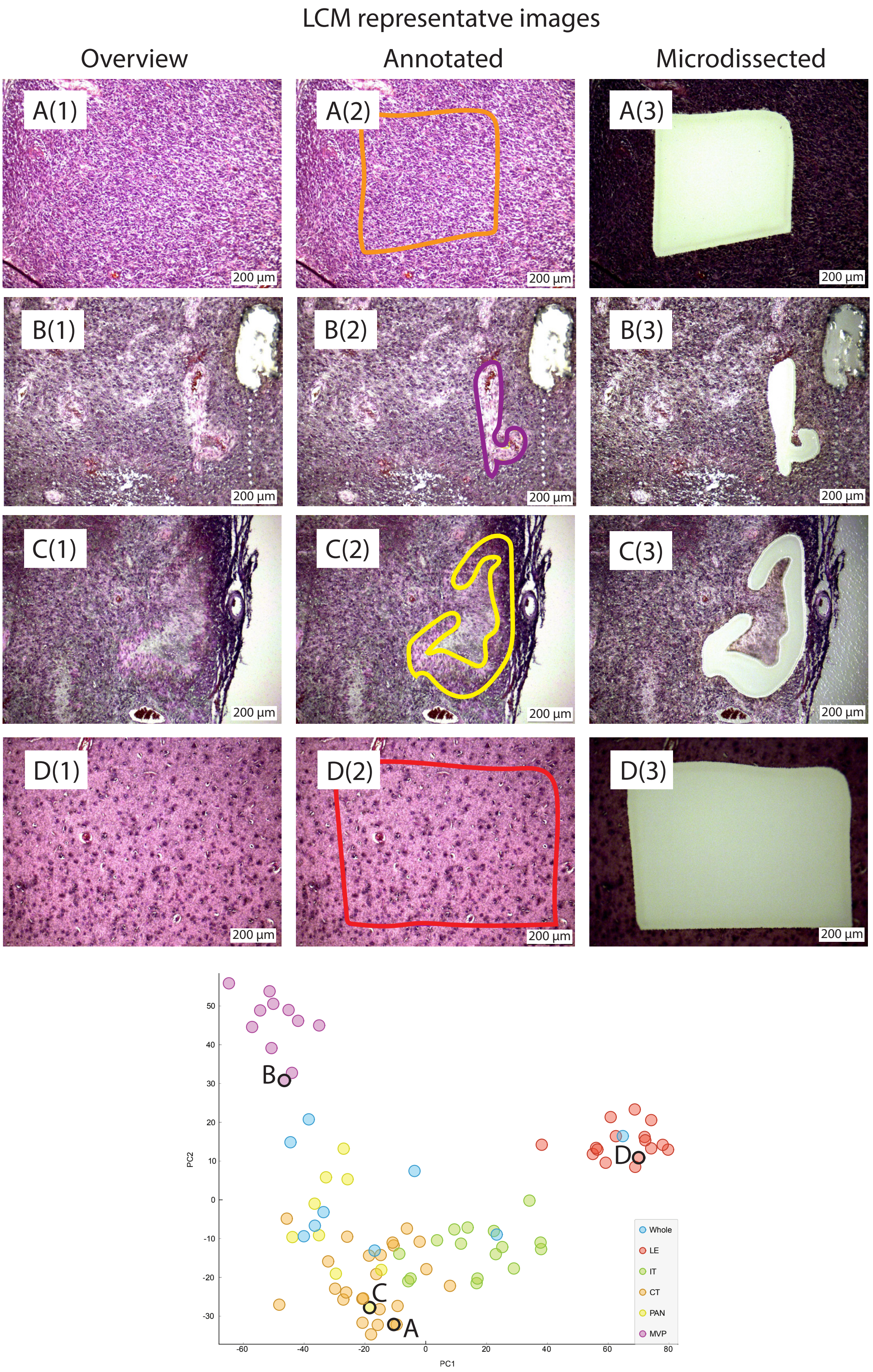

Fig S22. Patient 20 representative images before and after LMD
(**A**) CT representative images showing the overview (1), the annotation (2) and after microdissection (3)
(**B**) MVP representative images showing the overview (1), the annotation (2) and after microdissection (3)
(**C**) PAN representative images showing the overview (1), the annotation (2) and after microdissection (3)
(**D**) LE representative images showing the overview (1), the annotation (2) and after microdissection (3)

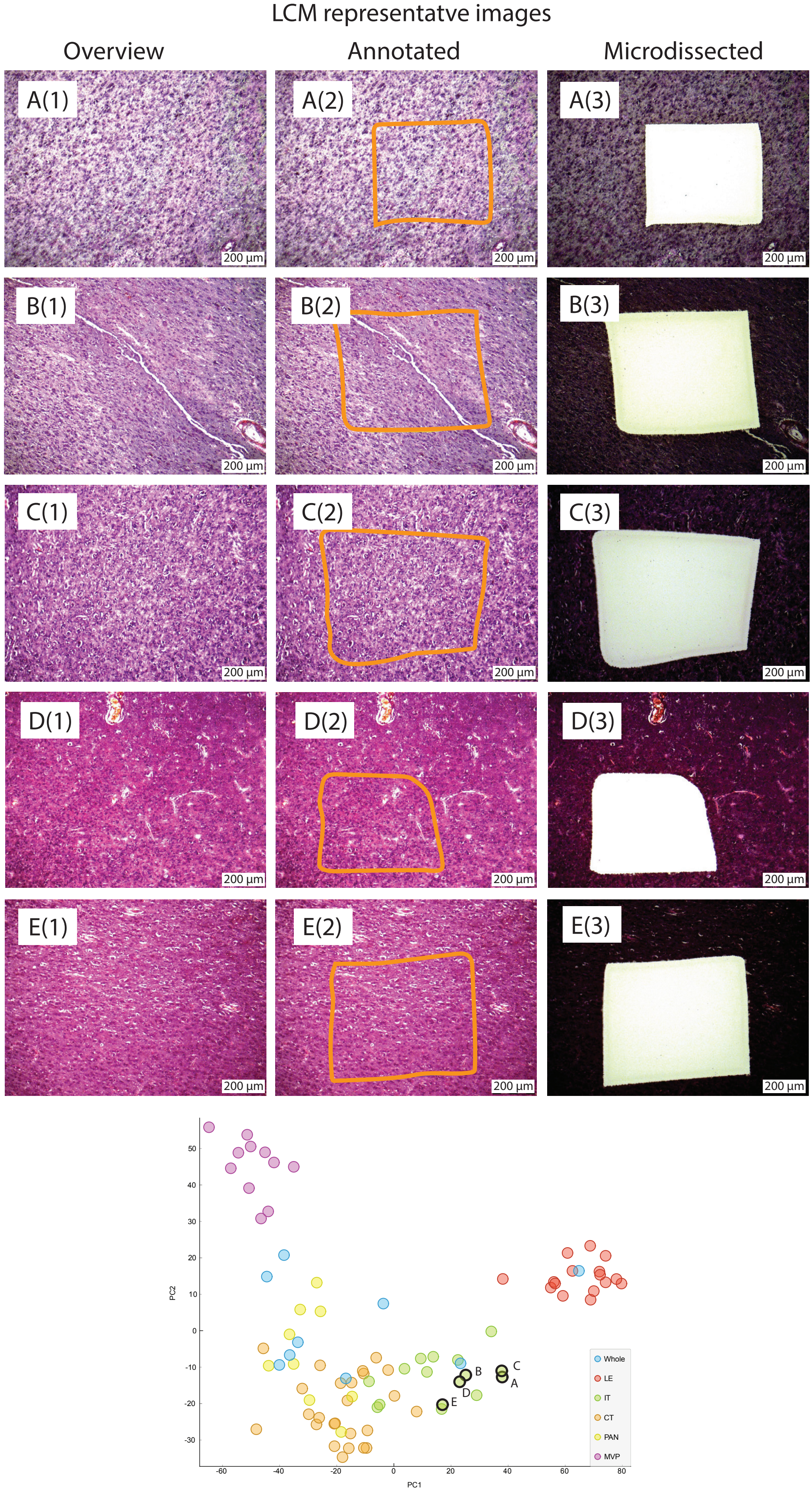

Fig S23. Patient 20 representative images before and after LMD
(**A**) IT representative images showing the overview (1), the annotation (2) and after microdissection (3)
(**B**) IT representative images showing the overview (1), the annotation (2) and after microdissection (3)
(**C**) IT representative images showing the overview (1), the annotation (2) and after microdissection (3)
(**D**) IT representative images showing the overview (1), the annotation (2) and after microdissection (3)
(**E**) IT representative images showing the overview (1), the annotation (2) and after microdissection (3)

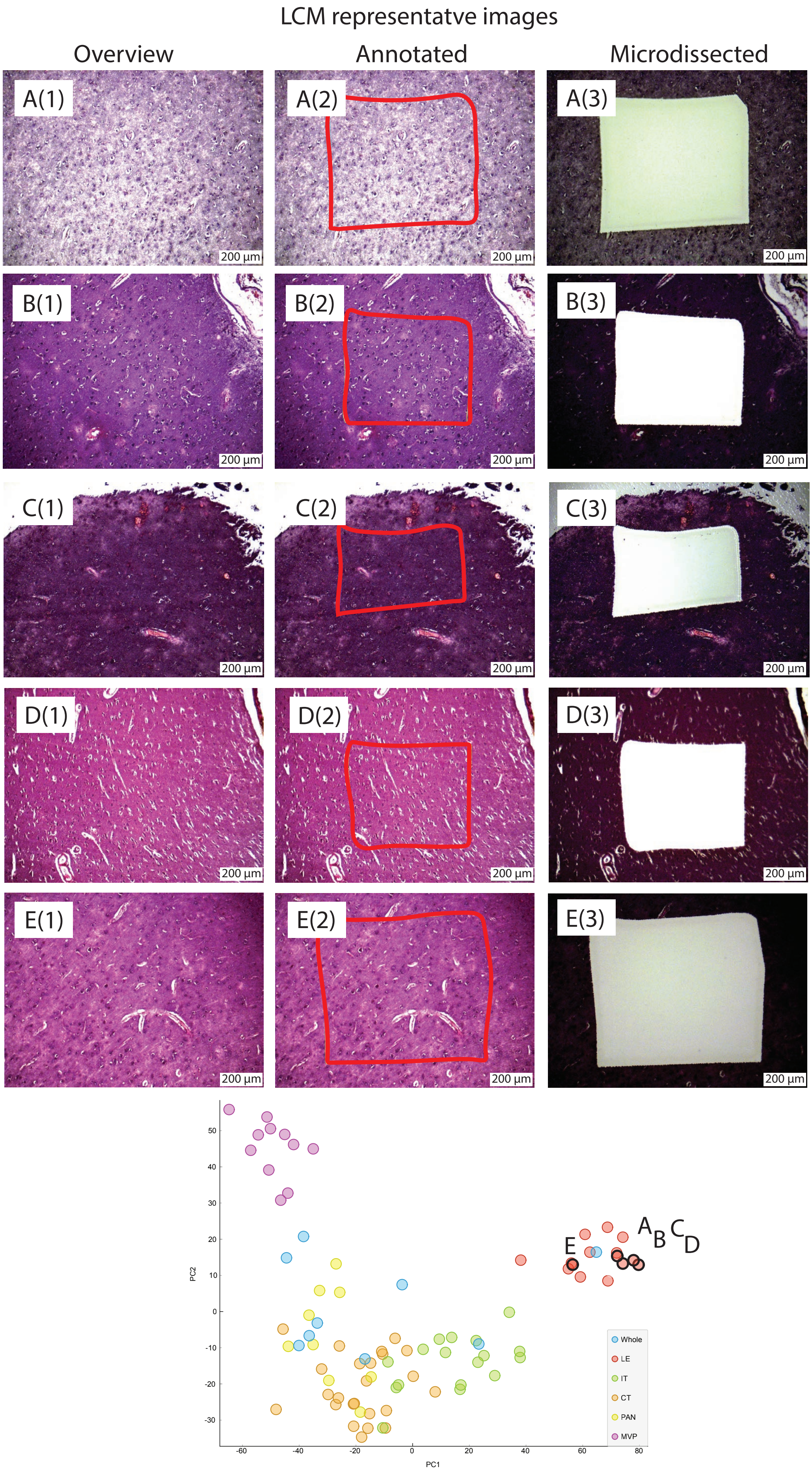

Fig S24. Patient 20 representative images before and after LMD

(**A**) LE representative images showing the overview (1), the annotation (2) and after microdissection (3)
(**B**) LE representative images showing the overview (1), the annotation (2) and after microdissection (3)
(**C**) LE representative images showing the overview (1), the annotation (2) and after microdissection (3)
(**D**) LE representative images showing the overview (1), the annotation (2) and after microdissection (3)
(**E**) LE representative images showing the overview (1), the annotation (2) and after microdissection (3)

Whole tissue representatve H & E images

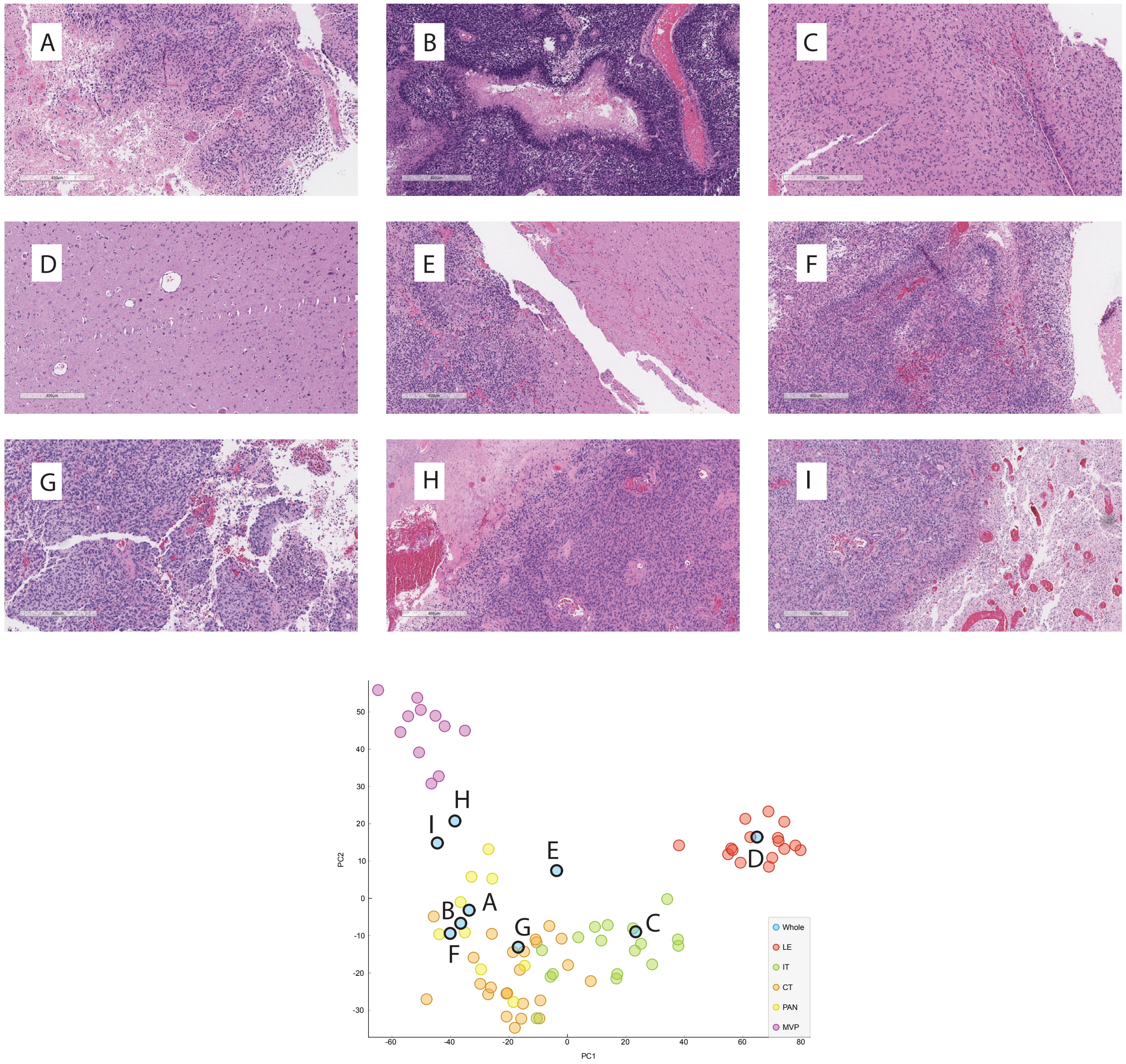

Fig S25. Whole tissue representative H & E images
(**A**) Patient 19 whole tumor section highlighting necrosis, PAN, CT and MVP. (**B**) Patient 13 whole tumor section highlighting necrosis, PAN, CT and MVP. (**C**) Patient 14 whole tumor section highlighting IT. (**D**) Patient 15 whole tumor section highlighting LE. (**E**) Patient 16 whole tumor section highlighting CT, MVP and LE. (**F**) Patient 1 whole tumor section highlighting PAN, CT and MVP. (**G**) Patient 5 whole tumor section highlighting CT, PAN and MVP. (**H**) Patient 11 whole tumor section highlighting necrosis, PAN, CT and MVP. (**I**) Patient 6 whole tumor section highlighting necrosis, PAN, CT and MVP.

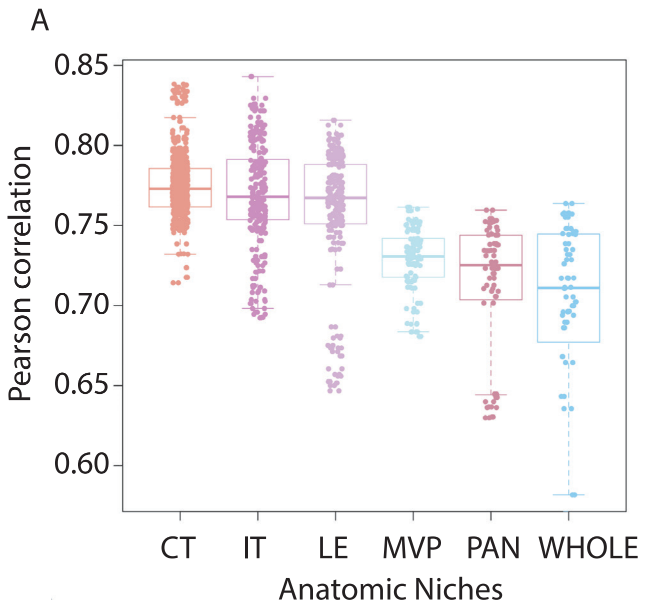

Figure S26. Box plot of pearson correlation coefficients

Box plots of the perason calculations were performed across all samples within a grouping based on all proteins and their relative abundances. (A) Pearson correlation coefficients comparisons across all samples (n=86 samples) within each histomorphological hallmark. Data are presented as mean values +/- SEM. The greatest variability is seen in WHOLE tissue samples as suggested by the low median pearson correlation and large interquartile range. (B) Pearson correlations across samples from the same patient and niche (n=57 samples). Data are presented as mean values +/- SEM. Overall the highmedian pearson correlation and small interquartile range suggest that samples from the same anatomical niche and patient have small differences and are generally governed by the same phenotypic processes.

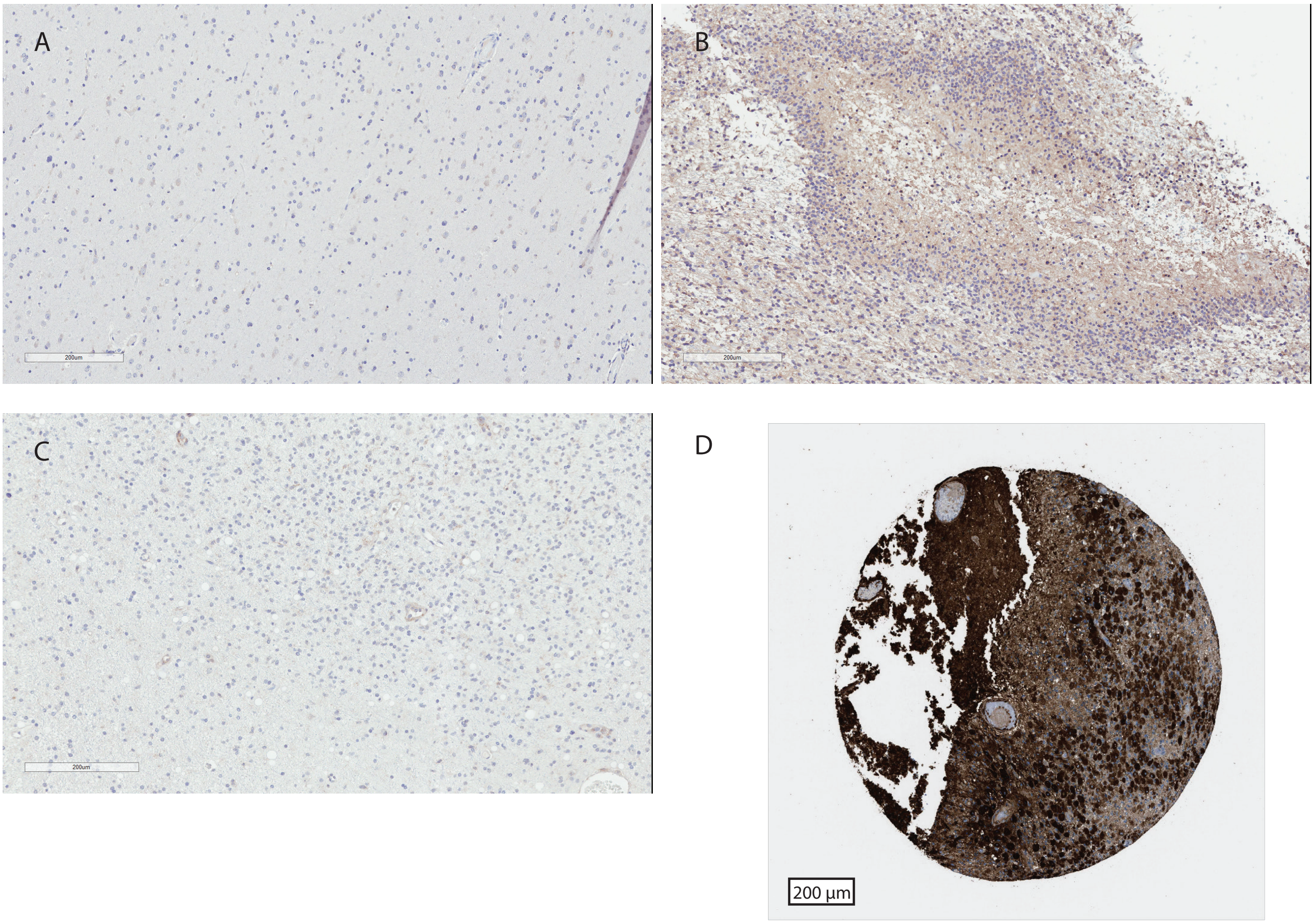

Figure S27. CD276 regional enrichment

Negative immunohistochemistry staining of CD276 in a (A) LE, (B) PAN and (C) IT regions. (D) Positive CD276 staining from the human protein atlas highlights heterogeneity in CD276 expression. The image can be accessed at [https://www.proteinatlas.org/ENSG00000103855-CD276/pathology/glioma#img][1].

1. Uhlén, M. et al. Tissue-based map of the human proteome. Science (80-.). 347, (2015).

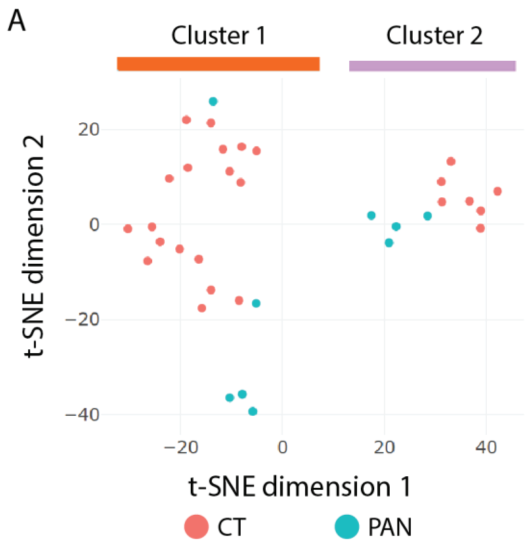

Figure S28. t-SNE

t-SNE dimensional reduction analysis of ssGSEA scores from 64 selected gene signatures (proteomics data) reveals the presence of two distinct clusters of samples independently of their histomorphometric niche.

A

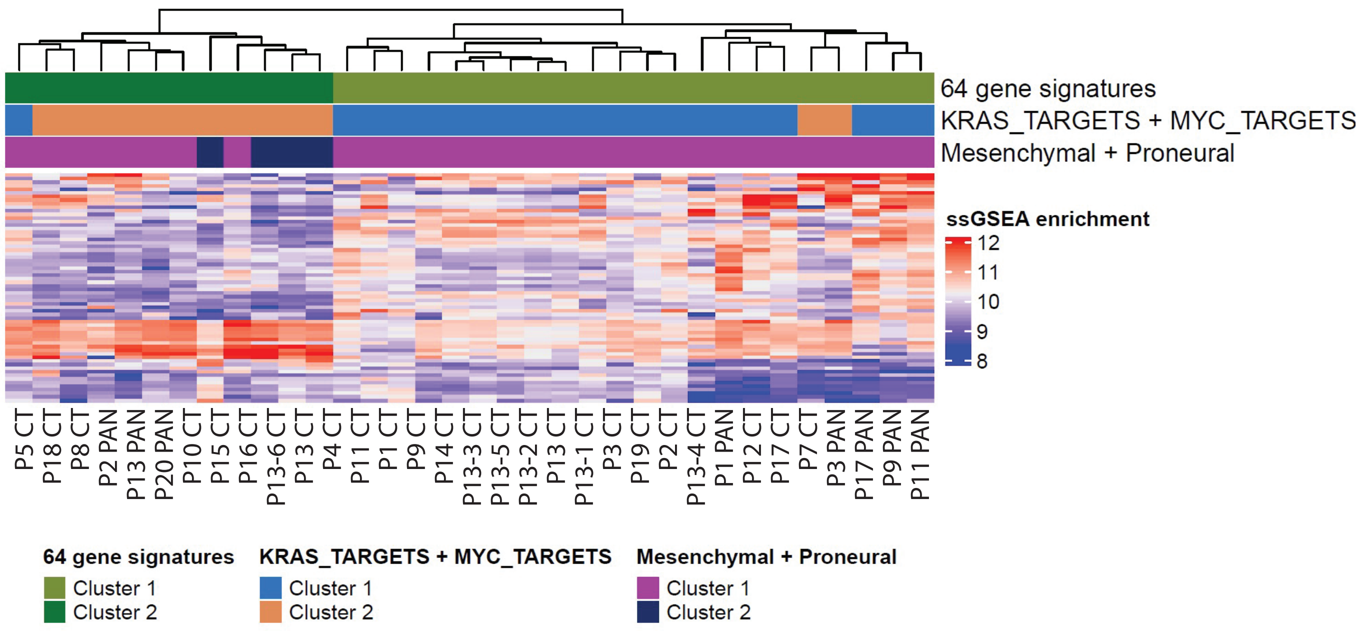

Figure S29. Comparison of the hierarchical clustering analyses

Comparison of the hierarchical clustering analyses that result from the use of different gene sets. The combination of the KRAS targets + MYC targets closesly matches the sample clusters obstained when using the 64 gene signatures (n=3), whereas the combination of the mesenchymal and proneural signatures produces a slightly higher number of mismatches (n=4).

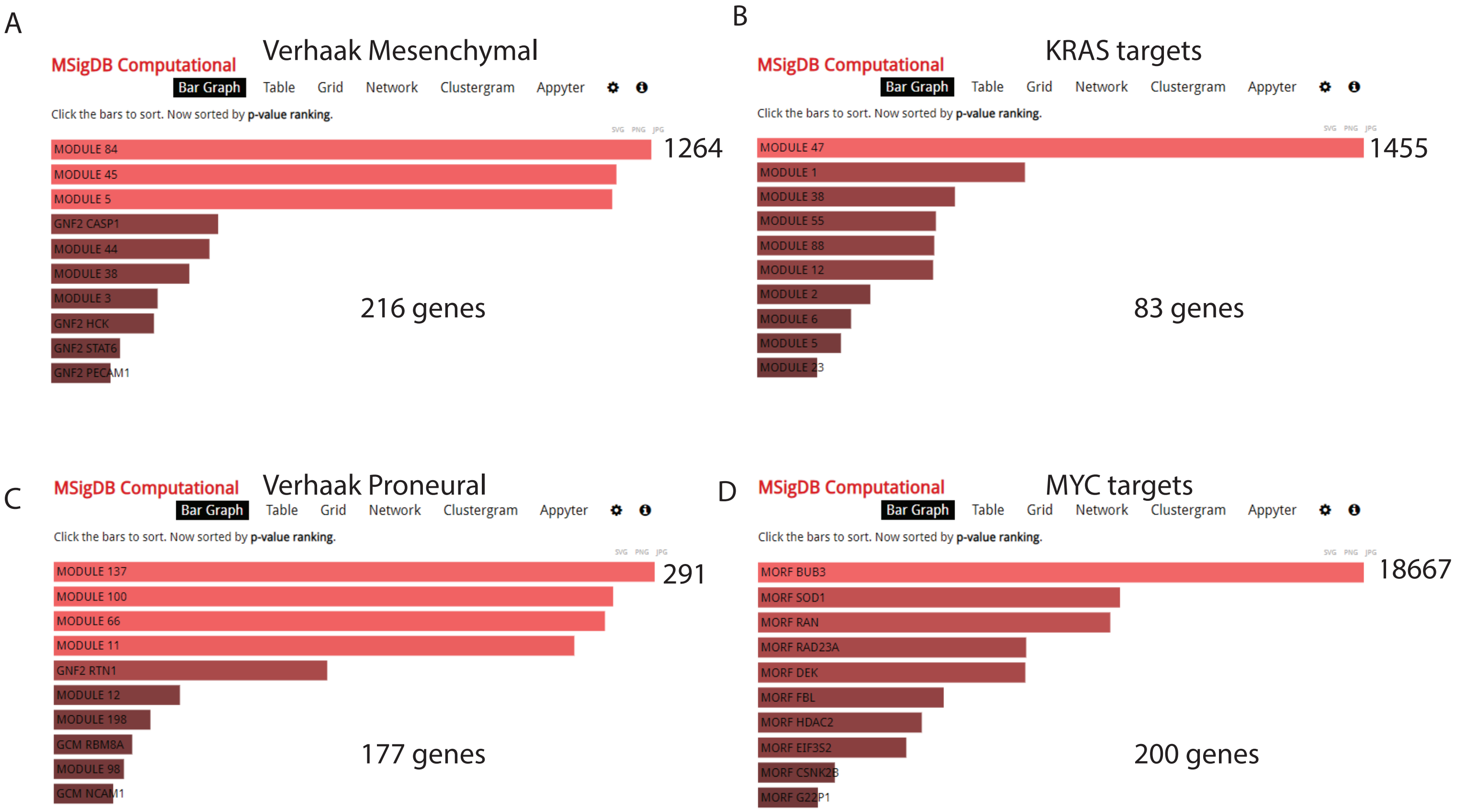

Figure S30. GSEA of pathway components for the MSigDB signatures. Pathway components of (A) Verhaak Mesenchymal, (B) KRAS targets, (C) Verhaak Proneural, and (D) MYC targets. The analysis show that the signatures KRAS_targets and MYC_targets are closely related to a single gene expression module, whereas the Verhaak signatures are influenced by multiple modules. The panels correspond to results produced by the online tool Enrichr (https://maayanlab.cloud/Enrichr/).

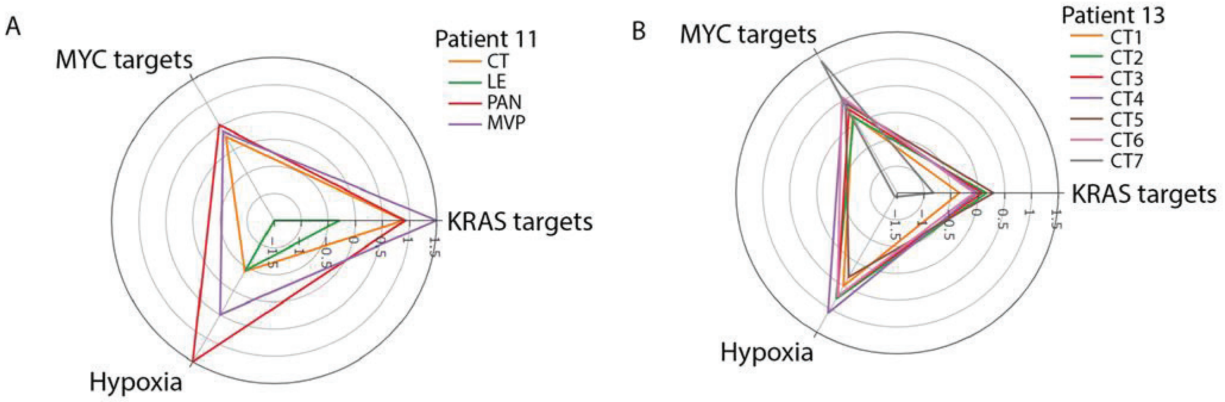

Figure S31. Functional status of histomorphometrically defined GBM niches in the context of our proposed triple-axis paradigm (proteomics data). (A) Representative example of several GBM niches within the same tumor. **(B)** Analysis of multiple CT tissue areas isolated from the same tumor specimen.

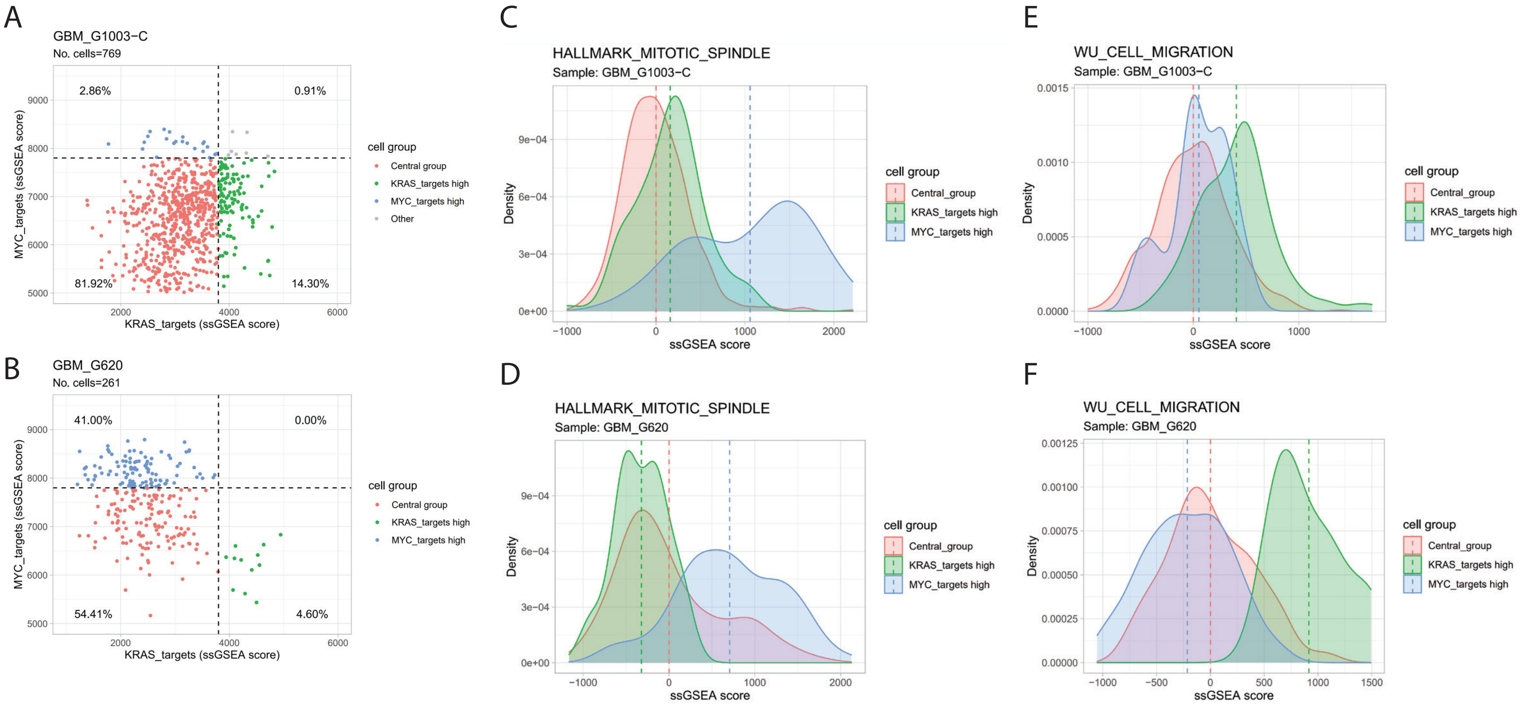

Figure S32. KRAS and MYC enrichment scores of cellular identities

Measurement of KRAS and MYC enrichment scores in scRNA data from Richards *et al.* from two tumors (A) G1003 (n=769 cells) and (B) G620 (n=261 cells). Subsequent GSEA of Hallmark Mitotic Spindle and Cell Migration processes highlighted that the (C,D) MYC cells were enriched for the Hallmark Mitotic Spindle pathway while the (E,F) KRAS group was enriched for the Cell Migration Pathway.

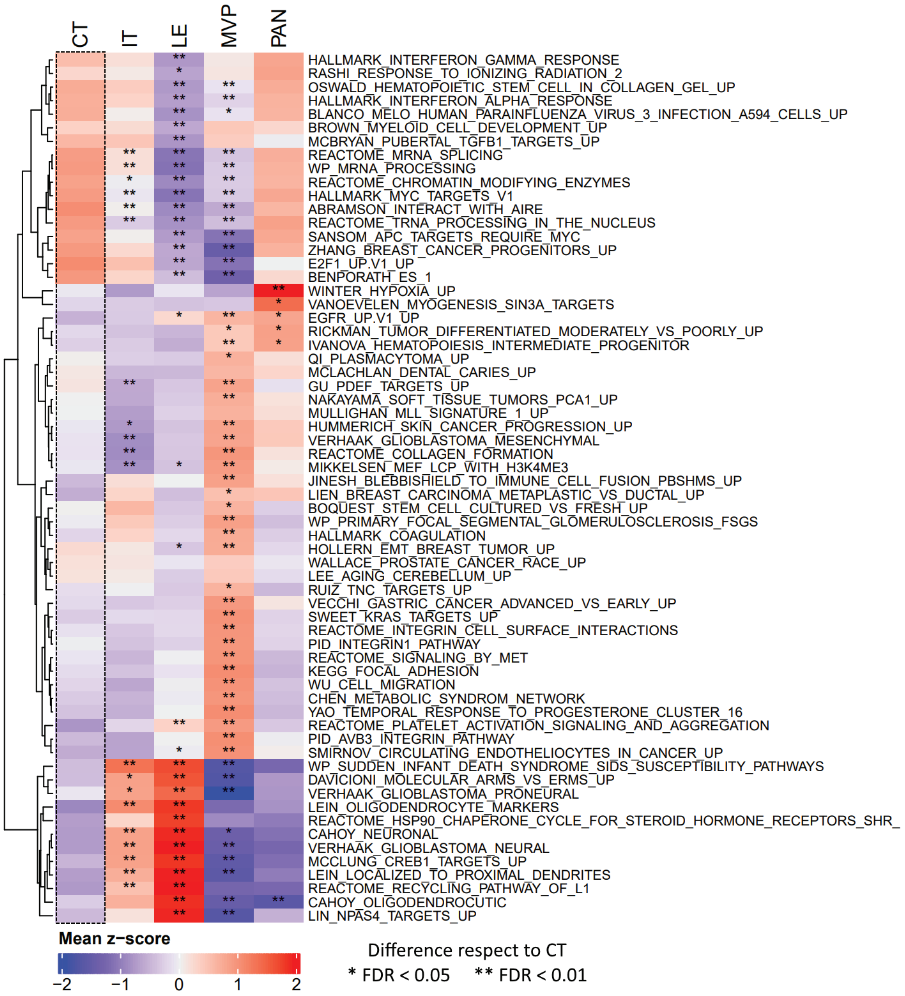

Figure S33. Relative levels of gene signature enrichment across histomorphologic groups.
ssGSEA scores for the 64 selected gene signatures were z-score normalized and for each signature, statistical differences between the mean values of IT, LE, MVP and PAN were calculated with respect to the CT group

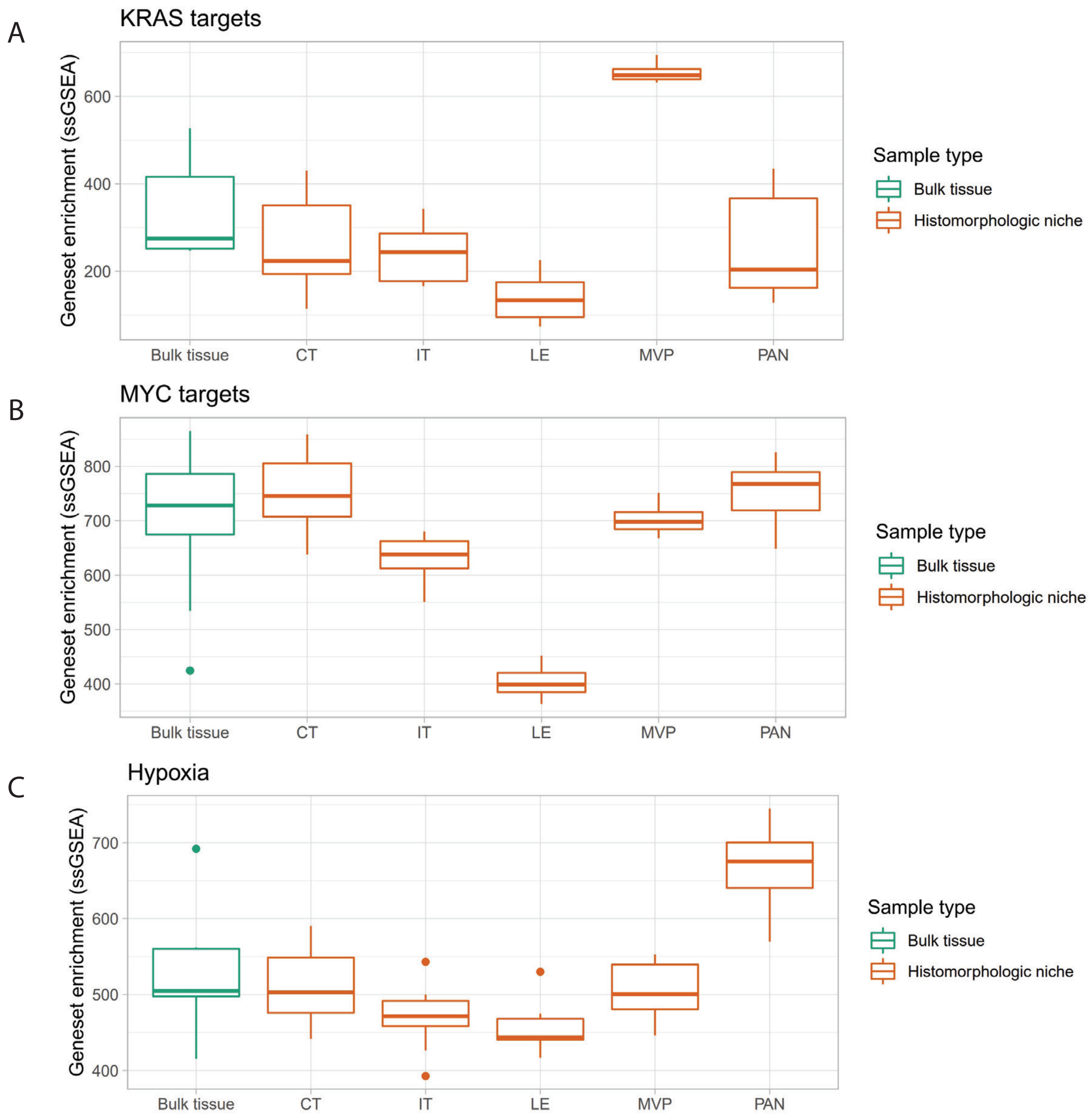

Figure S34. Gene signature enrichment of KRAS targets, MYC targets and hypoxia acorss GBM's hallmark histomorphologic niches and in whole tissue samples. The group of bulk samples (WHOLE) was analyzed by XGBoost inference for higher precision; since the samples belonging from defined histomorphologic groups were used to train and validate the XGBoost models, their ssGSEA ground truth is indicated. Gene set enrichment scores (ssGSEA) across histomorphological features were calculated for (A) KRAS targets, (B) MYC targets and (C) hypoxia (n=86) data are presented as the median value, the hinges correspond to the first and third quartiles, the top and bottom whiskers extend from the hinges to the maximum and minimum values within each quartile plus or minus 1.5 x IQR, respectively, and the dots correspond to outlier values as defined by the "1.5 rule".

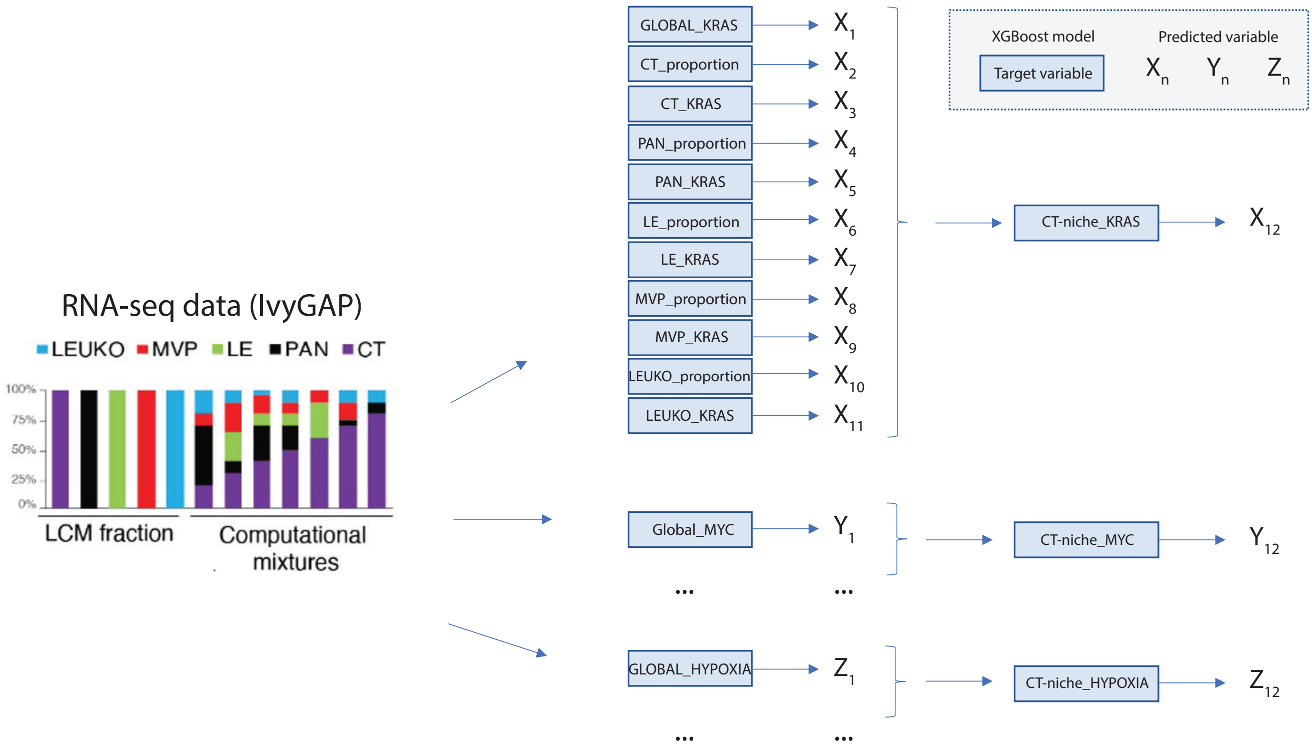

Figure S35. Schematic representation of the machine learning architecture for niche specific inference.

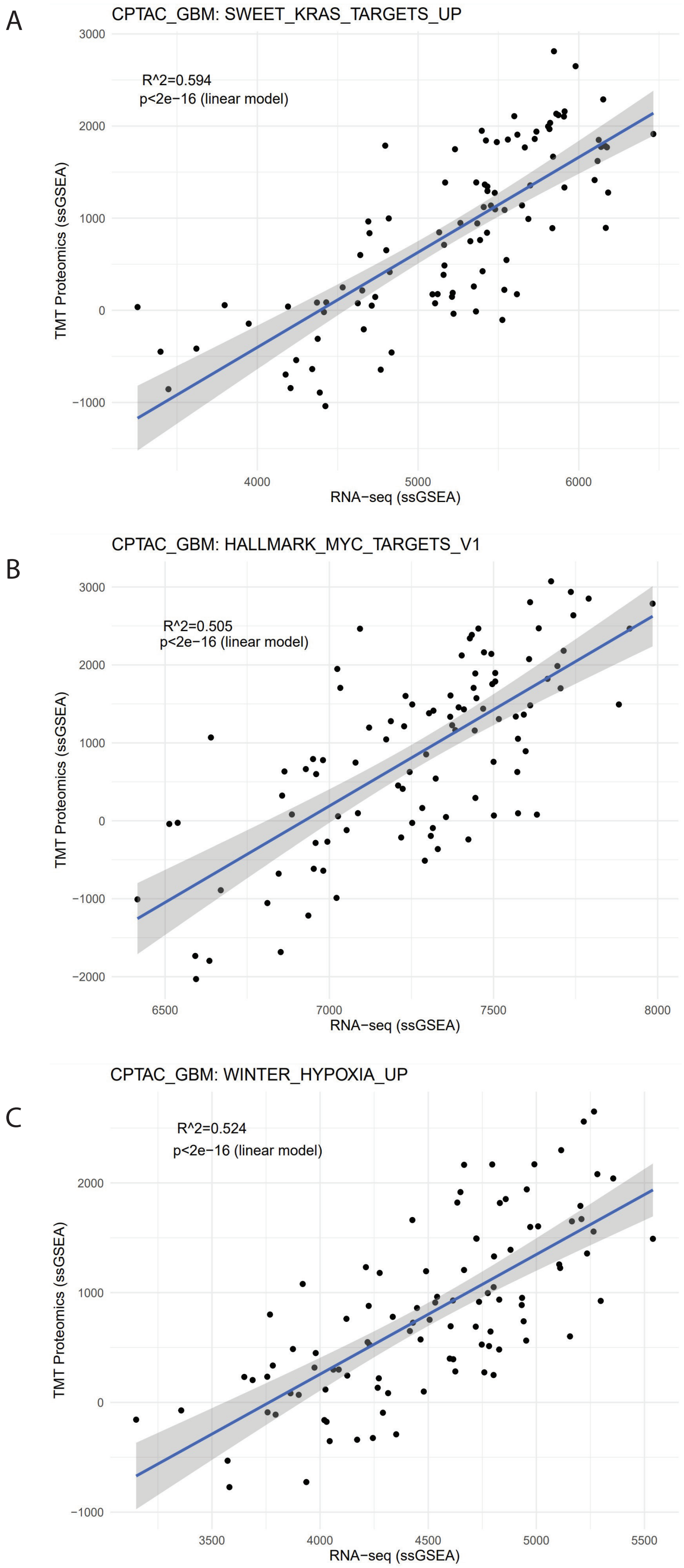

Figure S36. Concordance between RNA-seq and proteomics enrichment scores (ssGSEA) for the (A) KRAS_targets, (B) MYC_targets and (C) Hypoxia genesets in the CPTAC-GBM data (n=100). Null hypothesis testing was performed by permuted linear model fit analysis. The 95% confidence interval is shown as grey areas.

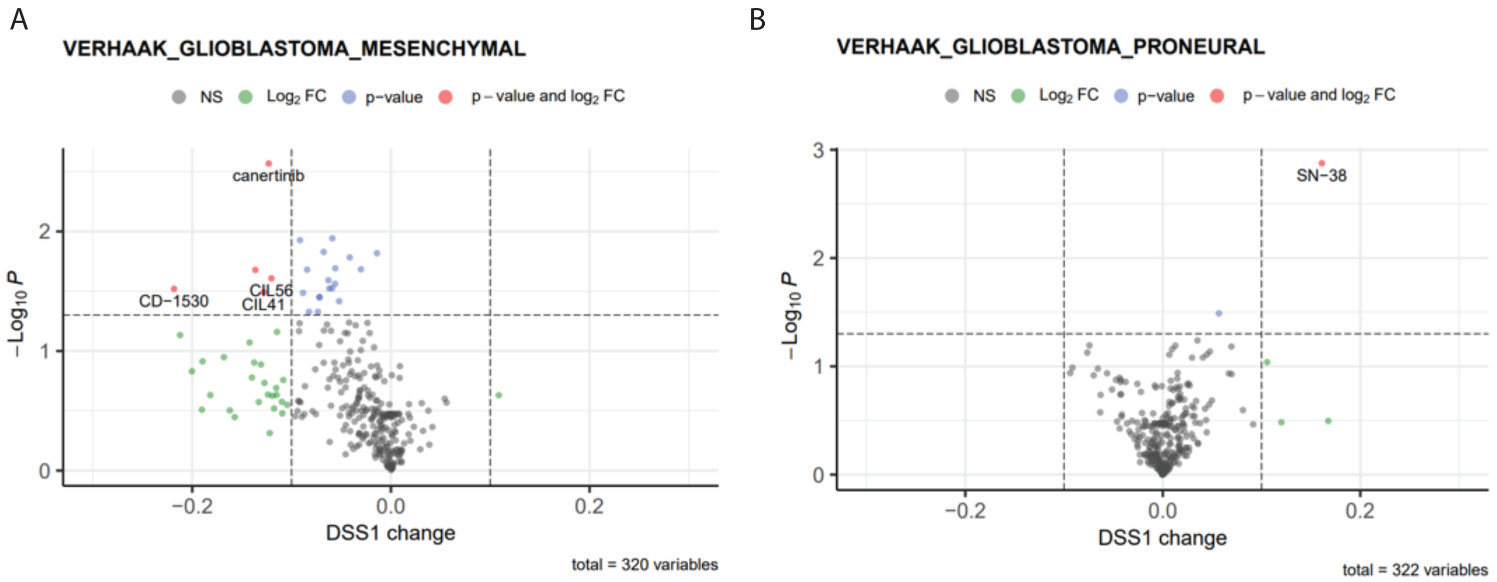

Figure S37. Pharmacological profiling highlights sensitivities of TCGA subtypes
Volcano plots showing pharmacotranscriptomic comparisons of drug sensitivities across cell lines ranking high and low (A) mesenchymal and (B) proneural signatures.

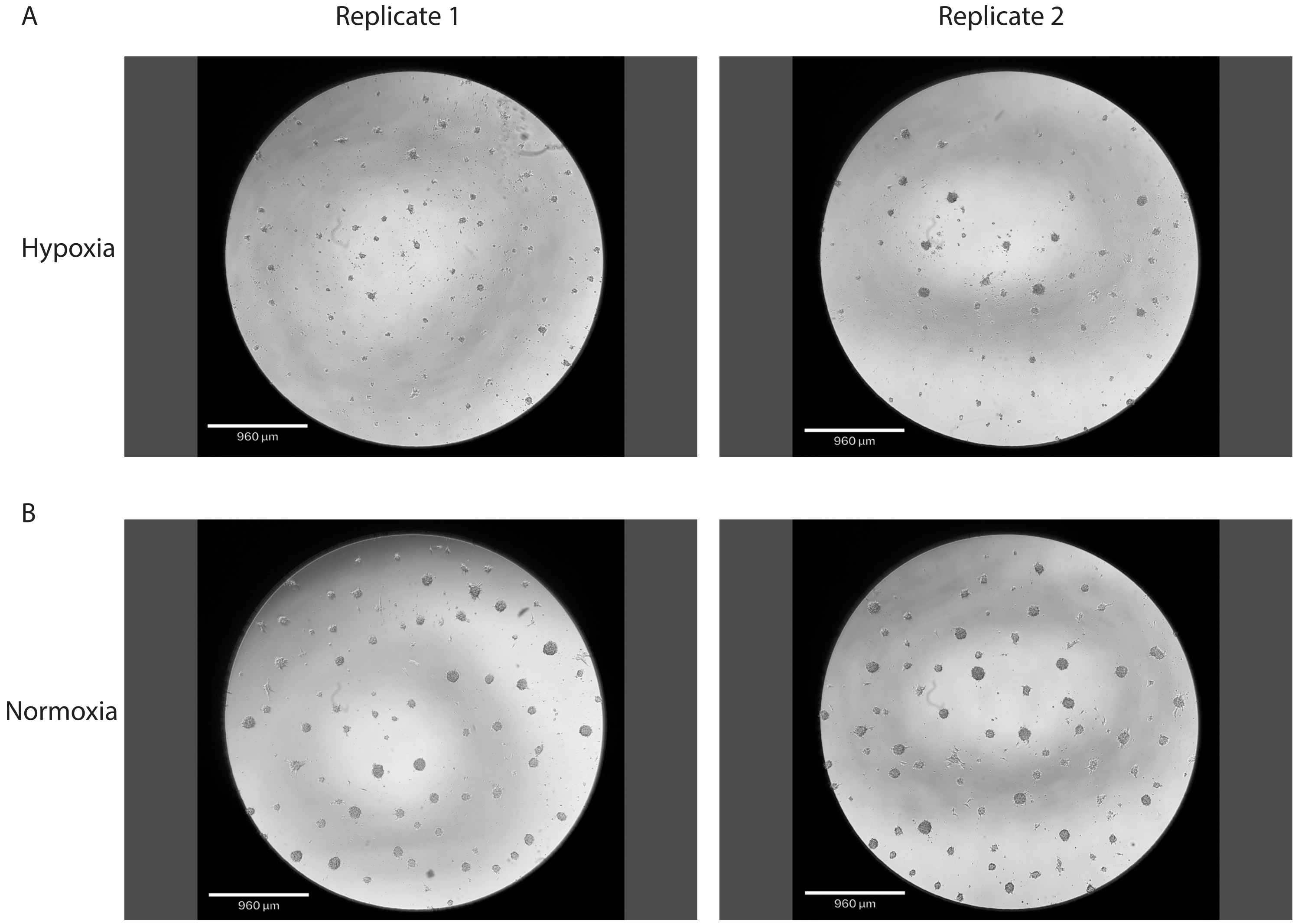

Figure S38. Spheroid images upon treatment with GSK949675

Relative viability effects on GSC proliferation in a kinome screen under differential oxygen concentrations. Spheroid images upon treatment of GSK949675, an AKT1, AKT2 and PRKCH inhibitor, in (A) hypoxic and (B) normoxic conditions in duplicate.
