# Supplementary material for: Topographic mapping of the glioblastoma proteome reveals a triple-axis model of intra-tumoral heterogeneity
Source: Nat Commun. 2022 Jan 10;13:116. doi: 10.1038/s41467-021-27667-w (PMC8748638; doi:10.1038/s41467-021-27667-w)
Supplement: Supplementary file 10 — Reporting Summary [file 41467_2021_27667_MOESM10_ESM.pdf]

nature research

Corresponding author(s): Phedias Diamandis

Last updated by author(s): Dec 3, 2021

Reporting Summary

Nature Research wishes to improve the reproducibility of the work that we publish. This form provides structure for consistency and transparency in reporting. For further information on Nature Research policies, see our Editorial Policies and the Editorial Policy Checklist.

Statistics

For all statistical analyses, confirm that the following items are present in the figure legend, table legend, main text, or Methods section.

n/a	Confirmed	
☐	☒	The exact sample size (*n*) for each experimental group/condition, given as a discrete number and unit of measurement
☐	☒	A statement on whether measurements were taken from distinct samples or whether the same sample was measured repeatedly
☐	☒	The statistical test(s) used AND whether they are one- or two-sided<br>*Only common tests should be described solely by name; describe more complex techniques in the Methods section.*
☐	☒	A description of all covariates tested
☐	☒	A description of any assumptions or corrections, such as tests of normality and adjustment for multiple comparisons
☐	☒	A full description of the statistical parameters including central tendency (e.g. means) or other basic estimates (e.g. regression coefficient) AND variation (e.g. standard deviation) or associated estimates of uncertainty (e.g. confidence intervals)
☐	☒	For null hypothesis testing, the test statistic (e.g. *F*, *t*, *r*) with confidence intervals, effect sizes, degrees of freedom and *P* value noted<br>*Give P values as exact values whenever suitable.*
☒	☐	For Bayesian analysis, information on the choice of priors and Markov chain Monte Carlo settings
☒	☐	For hierarchical and complex designs, identification of the appropriate level for tests and full reporting of outcomes
☐	☒	Estimates of effect sizes (e.g. Cohen's *d*, Pearson's *r*), indicating how they were calculated

Our web collection on statistics for biologists contains articles on many of the points above.

Software and code

Policy information about availability of computer code

Data collection	Leica LMD 7000 software was used to perform laser capture micro-dissection and collect anatomical samples. Xcalibur software 4.3 was used for the acquisition of mass-spectrometry-based proteomics data.
Data analysis	Mass spectrometry raw data files were processed using MaxQuant Andromeda search engine v1.6.17.0 (www.coxdocs.org) against the Human Swissprot protein database (July, 2019 version). Analysis of proteomic data was performed using a variety of biostatistical platforms Perseus 1.6.15.0 (www.coxdocs.org), R 4.0.4 (www.r-project.org), Orange 3.27.1 (https://orange.biolab.si/), GSEA 4.1.0 (https://www.gsea-msigdb.org/gsea/index.jsp), ssGSEA v4, Cytoscape 3.9.0, and XGBoost 1.4.1.1. Heatmaps were generated using the ComplexHeatmap 2.9.3 package (http://bioconductor.org/packages/release/bioc/html/ComplexHeatmap.html). Gene set enrichment scores at the cell level were calculated using the gene set variation analysis package GSVA 4.1(10.18129/B9.bioc.GSVA). RNA-seq reads were processed using STAR v2.6.0c and quantified using RSEM v1.3.0. Drug sensitivity data of the dataset "CTRPv2" was obtained from the PharmacoGx package 3.13 (https://bioconductor.org/packages/release/bioc/html/PharmacoGx.html). Activation of the 64 gene signatures can be found at (https://github.com/diamandis-lab/paper-prot-atlas-gbm)

For manuscripts utilizing custom algorithms or software that are central to the research but not yet described in published literature, software must be made available to editors and reviewers. We strongly encourage code deposition in a community repository (e.g. GitHub). See the Nature Research guidelines for submitting code & software for further information.

Data

Policy information about availability of data

All manuscripts must include a data availability statement. This statement should provide the following information, where applicable:

- Accession codes, unique identifiers, or web links for publicly available datasets
- A list of figures that have associated raw data
- A description of any restrictions on data availability

The anatomical atlas of human glioblastoma mass spectrometry proteomics data have been deposited to the ProteomeXchange Consortium via the PRIDE partner repository with the dataset identifier PXD019381 [https://www.ebi.ac.uk/pride/archive/projects/PXD019381]. The XGBoost mass spectrometry proteomics data have been deposited to the ProteomeXchange Consortium via the PRIDE partner repository with the dataset identifier PXD029290 [https://www.ebi.ac.uk/pride/archive/projects/PXD029290]. The RNA data generated in this study have been deposited in the Zenodo database under accession code 10.5281/zenodo.5639569 [https://doi.org/10.5281/zenodo.5639569]. The RNA-seq dataset was deposited in the European Genome-Phenome Archive with the accession identifier EGAD00001008362 [https://ega-archive.org/datasets/EGAD00001008362]. Data used in this publication were generated by the Clinical Proteomic Tumor Analysis Consortium (NCI/NIH) and are publicly available [https://cptac-data-portal.georgetown.edu/study-summary/S048]. Data used in this publication were generated by The Cancer Genome Atlas Program (TCGA) and deposited at the Data Coordinating Center (DCC) for public access [http://cancergenome.nih.gov/]. The IvyGAP data including the RNA-Seq and copy number data are publically available at Gene Expression Omnibus through GEO series accession number GSE107560. The single cell are publicly available through the Broad Institute Single-Cell Portal (https://singlecell.broadinstitute.org/single_cell/study/SCP503) and CReSCENT60 (https://crescent.cloud; study ID CRES-P23). The data generated in this study are provided in the Supplementary Information/Source Data file. RNA-seq data and drug sensitivities from 33 glioblastoma cell lines from the Cancer Cell Line Encyclopedia is accessible at (https://portals.broadinstitute.org/ccle). The MSigDB database can be accessed at (https://www.gsea-msigdb.org/gsea/index.jsp). Source data are provided with this paper.

Field-specific reporting

Please select the one below that is the best fit for your research. If you are not sure, read the appropriate sections before making your selection.

☒ Life sciences ☐ Behavioural & social sciences ☐ Ecological, evolutionary & environmental sciences

For a reference copy of the document with all sections, see nature.com/documents/nr-reporting-summary-flat.pdf

Life sciences study design

All studies must disclose on these points even when the disclosure is negative.

Sample size	No sample-size calculation was performed. For mass spectrometry analysis, we utilized a cohort of 20 patients with 5 profiled niches per patient (resulting in n~20 samples per region). These samples were derived after curation of tissue by neuropathologists as representative glioblastoma tumors. This provided sufficient statistical power to provide region specific putative biomarkers that could be orthogonally validated by immuno-histochemistry.
Data exclusions	For proteomic analysis, the number of proteins identified per sample were used to determine sample outliers. Outlier analysis (cook's distance and leverage plots) were performed utilizing the MaxQuant output on the number of proteins identified by MS/MS and the total number of proteins identified. Samples that were identified as outliers were removed from downstream analyses. These samples were removed because they did not have the same number of identified proteins and would bias the statistical analyses.
Replication	Mass-spectrometry based proteomics analysis was performed in duplicate and their reproducibility was analyzed by the number of proteins identified for each duplicate. Samples that were not reproducible were identified by outlier analysis (cook's distance and leverage plots) due to the stochastic nature of mass spectrometry. Non-reproducible samples were removed from further analyses.
Randomization	All samples were anonymized throughout the study, niche and regional samples were randomized throughout the analytical portion of the study.
Blinding	Selection of anatomical niches, for each tumor was independently performed by each neuropathologist. Their selection of anatomical niches were blinded to each other, and only the independently agreed upon selections were utilized. During data collection, investigators were not blinded because investigators needed to identify specific regions of the tumors for collection. Blinding is not relevant to this study, as tumor regions are of biological relevance.

Reporting for specific materials, systems and methods

We require information from authors about some types of materials, experimental systems and methods used in many studies. Here, indicate whether each material, system or method listed is relevant to your study. If you are not sure if a list item applies to your research, read the appropriate section before selecting a response.

Materials & experimental systems

n/a	Involved in the study	
☐	☒	Antibodies
☐	☒	Eukaryotic cell lines
☒	☐	Palaeontology and archaeology
☒	☐	Animals and other organisms
☐	☒	Human research participants
☒	☐	Clinical data
☒	☐	Dual use research of concern

Methods

n/a	Involved in the study	
☒	☐	ChIP-seq
☒	☐	Flow cytometry
☒	☐	MRI-based neuroimaging

Antibodies

Antibodies used	anti-CD276, Sigma-Aldrich, HPA009285, 10150251, 1:25; anti-AKAP12, Sigma-Aldrich, HPA006344, B81315, 1:75; anti-PTPRZ1, Sigma-Aldrich, HPA015103, 1:600; anti-SLC2A1, Sigma-Aldrich, HPA031345,1:500; anti-SERPINH1, Sigma-Aldrich, HPA029198, 1:20; anti-CA9, Novus Biologicals, NBP1-51691, 1:1000; Rabbit-HRP, Dako, K400311-2, 10150251, no dilution.
Validation	All primary antibodies have immuno-histochemistry profiles that can be seen in the Human Protein Atlas (HPA) project (www.proteinatlas.org) across 44 normal human tissues and 20 of the most common cancer tissues. Anti-CD276, orthogonal RNAseq recombinant expression; anti-AKAP12, independent orthogonal RNAseq; anti-PTPRZ1, orthogonal RNAseq; ANTI-SLC2A1, orthogonal RNAseq; anti-SERPINH1, RNAi knockdown; anti-CA9, orthogonal RNAseq.

Eukaryotic cell lines

Policy information about cell lines

Cell line source(s)	All cell lines are derived from human tumor.
Authentication	RNAseq confirmed pre-existing tumor mutations and mouse xenotransplantation experiments confirmed GBM forming potential.
Mycoplasma contamination	All cell lines tested negative for mycoplasma contamination
Commonly misidentified lines (See ICLAC register)	n/a

Human research participants

Policy information about studies involving human research participants

Population characteristics	Cohort characteristics in age, gender, genotypic information, and diagnosis were collected for each patient.
Recruitment	Tissue samples used in this study were retrieved from retrospective archival specimens from the pathology department and related tumor tissue banks.
Ethics oversight	Patient consent was not directly obtained and a consent waiver for this study was granted by the University Health Network Research Ethics Board as the research was deemed to involve no more than a minimal risk as it included use of exclusively existing pathology specimens.

Note that full information on the approval of the study protocol must also be provided in the manuscript.
